# MicroRNA Profiling of Primary Cutaneous Large B-Cell Lymphomas

**DOI:** 10.1371/journal.pone.0082471

**Published:** 2013-12-16

**Authors:** Lianne Koens, Yongjun Qin, Wai Y. Leung, Willem E. Corver, Patty M. Jansen, Rein Willemze, Maarten H. Vermeer, Cornelis P. Tensen

**Affiliations:** 1 Department of Pathology, Leiden University Medical Center, Leiden, The Netherlands; 2 Department of Dermatology, Leiden University Medical Center, Leiden, The Netherlands; 3 Biotechnology Center, Shanxi Academy of Agricultural Sciences, Taiynan, China; IPMC, CNRS UMR 7275 UNS, France

## Abstract

Aberrant expression of microRNAs is widely accepted to be pathogenetically involved in nodal diffuse large B-cell lymphomas (DLBCLs). However, the microRNAs profiles of primary cutaneous large B-cell lymphomas (PCLBCLs) are not yet described. Its two main subtypes, i.e., primary cutaneous diffuse large B-cell lymphoma, leg type (PCLBCL-LT) and primary cutaneous follicle center lymphoma (PCFCL) are characterized by an activated B-cell (ABC)-genotype and a germinal center B-cell (GCB)-genotype, respectively. We performed high-throughput sequencing analysis on frozen tumor biopsies from 19 cases of PCFCL and PCLBCL-LT to establish microRNA profiles. Cluster analysis of the complete microRNome could not distinguish between the two subtypes, but 16 single microRNAs were found to be differentially expressed. Single microRNA RT-qPCR was conducted on formalin-fixed paraffin-embedded tumor biopsies of 20 additional cases, confirming higher expression of miR-9-5p, miR-31-5p, miR-129-2-3p and miR-214-3p in PCFCL as compared to PCLBCL-LT. MicroRNAs previously described to be higher expressed in ABC-type as compared to GCB-type nodal DLBCL were not differentially expressed between PCFCL and PCLBCL-LT. In conclusion, PCFCL and PCLBCL-LT differ in their microRNA profiles. In contrast to their gene expression profile, they only show slight resemblance with the microRNA profiles found in GCB- and ABC-type nodal DLBCL.

## Introduction

Primary cutaneous large B-cell lymphomas (PCLBCLs) are a group of malignant lymphoproliferative disorders presenting in the skin with no evidence of extracutaneous disease at the time of diagnosis [1]. In the latest World Health Organization (WHO) 2008 classification two main types of PCLBCL are distinguished: primary cutaneous follicle center lymphoma (PCFCL) and primary cutaneous diffuse large B-cell lymphoma, leg type (PCLBCL-LT) [2]. PCFCL is considered an indolent type of lymphoma (5 year overall survival (OS) > 95%), whereas PCLBCL-LT has a more aggressive clinical course (5 year OS approximately 40%) [3]. The two subtypes also show marked differences at a molecular level by array comparative genomic hybridization [4] and gene expression profiling. PCFCL and PCLBCL-LT have gene expression profiles corresponding to germinal center B-cell (GCB)-type diffuse large B-cell lymphoma (DLBCL) and activated B-cell (ABC)-type nodal DLBCL, respectively [5]. Clinical distinction at the time of first diagnosis between these different subtypes of PCLBCL is important, as the first choice of treatment differs between the two entities. PCFCL can be adequately treated with radiotherapy, but the more aggressive behavior of PCLBCL-LT warrants for treatment with anthracyclin-based chemotherapy combined with rituximab [6].

MicroRNAs are approximately 22 nucleotides long non-coding RNA molecules that can regulate translation of several specific target messenger RNAs (mRNAs). The two major responsible mechanisms are inhibition of translational initiation by blocking ribosomes and direct targeting of mRNA at the 3 prime untranslated region leading to degradation of the molecule [7]. Alterations in the expression of microRNAs contribute to the pathogenesis of different types of malignancies, including malignant lymphomas, but are also involved in normal development of hematopoietic cells [8]. Although a role for microRNAs in the pathogenesis of nodal DLBCLs is generally recognized, little is known about the presence and significance of microRNAs in PCLBCL. In one recently published study, a limited set of microRNAs was investigated in PCFCL [9].

In nodal DLBCL, much effort has been given to differentiate between the two major molecular subtypes, i.e. GCB-type DLBCL and ABC-type or non-GCB-type DLBCL, by means of expression of specific microRNAs or microRNA profiles. However, conflicting results concerning this topic have been published. The most frequently encountered microRNA alterations are upregulation of miR-155, miR-21, miR-221 and miR-222 in ABC-type DLBCL as compared to GCB-type DLBCL as detected by microarray profiling and/or Real Time quantitative PCR (RT-qPCR) [10]–[19]. To date, the prognostic value of expression levels of specific microRNAs in nodal DLBCL remains unclear. Although higher expression of miR-222 correlating to a shorter progression-free survival (PFS) was reproduced in more than one study [12], [15], [20], a correlation was not consistently found for other microRNAs. In addition, reported microRNA profiles generated by complete microRNA profiling that would distinguish between the two subtypes of nodal DLBCL have no substantial similarities (Table S1) [12], [14]–[18], [21]. To some extent, the observed variations might be explained by the different techniques used to quantify microRNAs, i.e. different microarrays or varying reference RNAs in RT-PCR experiments. Furthermore, the techniques used to distinguish the two molecular subtypes from each other are not uniform. The encountered differences might on the other hand also reflect the profound tumor heterogeneity within the group of nodal DLBCLs.

In search of differences in the pathogenesis of PCFCL and PCLBCL-LT we investigated the microRNA profiles in PCLBCL by performing microRNA high-throughput sequencing. Several single microRNAs that were differentially expressed between PCFCL and PCLBCL-LT in our high-throughput sequence data were validated by RT-qPCR. Furthermore, we were interested in comparing the microRNA profiles of PCFCL and PCLBCL-LT with the (known) microRNA profiles of GCB- and ABC-type nodal DLBCL, in search for common pathogenetical pathways and common targets for tailored therapy.

## Materials and Methods

### Tumor samples

Frozen skin biopsy or excision material from primary tumors was collected from 6 patients with PCFCL and 13 patients with PCLBCL-LT from the archives of the Leiden University Medical Center (Leiden, The Netherlands). Formalin-fixed and paraffin-embedded (FFPE) skin biopsy material was available from 5 of these patients (2 PCFCL and 3 PCLBCL-LT cases) and an additional 8 patients with PCFCL and 7 patients with PCLBCL-LT. The diagnosis was based on the criteria of the WHO-EORTC and WHO 2008 classifications [1], [22], and confirmed by a panel of hematopathologists and dermatologists, aided by several additional immunohistochemical stainings. The clinical data and expression of relevant immunohistochemical markers are summarized in Table 1. In all cases physical examination, computed tomography scan, bone marrow examination and peripheral blood counts did not show extracutaneous disease. Furthermore, FFPE lymph node excisions of 34 cases of nodal DLBCL were collected from the Leiden University Medical Center, the Diaconessenhuis (Leiden, The Netherlands) and the Reinier de Graaf Hospital (Delft, The Netherlands). Of those, 19 were ABC-type and 11 were GCB-type, according to both Hans and Choi immunohistochemical algorithms [23], [24], the four samples with discrepancy between the two algorithms were discarded from further analysis. The tumor cell percentage was at least 75% in every tumor specimen, as shown by immunohistochemistry for CD20 and CD3. All tissue samples were handled in a coded fashion, according to the Dutch National Ethical guidelines (Code for Proper Secondary Use of Human Tissue, Dutch Federation of Medical Scientific Societies), waving the need for specific approval of the study by the ethical committee and patient informed consent.

**Table 1:** Patient characteristics.

Case	Experiment	Sex	Age at diagnosis	Primary tumor	Immunohistochemistry					Initial therapy	Recurrence	PFS, months	Follow-up, months	Current status
					BCL2	BCL6	CD10	IRF4	BCR					
Primary cutaneous follicle center lymphoma														
1	HTS & RT-PCR	Female	81	Head	-	+	-	-	-	RT	Yes	62	86	A^0^
2	HTS & RT-PCR	Male	71	Trunk	-	+	-	-	-	RT + oral prednisone	No	89	90	A^0^
3	HTS	Female	71	Head	-	+	-	-	-	RT	No	55	56	A^0^
4	HTS	Male	72	Head	+	+	-	-	-	RT	No	32	33	A^0^
5	HTS	Male	67	Head	-	+	-	-	*NA*	RT	No	55	56	A^0^
6	HTS	Male	59	Arm	-	+	-	-	IgM, IgD	RT	Yes	8	9	A^+^
7	RT-PCR	Female	53	Head	-	+	-	-	IgM, IgD, λ	RT	Yes	46	128	A^0^
8	RT-PCR	Male	33	Trunk	+	+	-	-	-	RT	Yes	60	185	A^+^
9	RT-PCR	Male	55	Trunk	-	*NA*	-	-	-	RT	Yes	5	105	A^0^
10	RT-PCR	Female	50	Trunk	-	+	-	-	-	RT	No	63	64	A^0^
11	RT-PCR	Male	47	Trunk	-	+	-	-	-	RT	Yes	7	68	A^+^
12	RT-PCR	Male	44	Head	-	+	-	-	-	RT	No	52	53	A^0^
13	RT-PCR	Male	35	Trunk	-	+	-	-	-	RT + PCT	No	20	26	A^0^
14	RT-PCR	Male	66	Trunk	-	+	-	-	-	RT	Yes	11	36	A^+^
Primary cutaneous large B-cell lymphoma, leg type														
1	HTS & RT-PCR	Male	88	Leg	+	+	-	+	IgM, IgD, κ	PCT	Yes	2	12	D^+^
2	HTS & RT-PCR	Male	81	Leg	+	+	-	+	IgM, IgD, λ	None	No	0	2	D^+^
3	HTS & RT-PCR	Male	68	Leg	+	+	-	+	IgM, IgD, λ	PCT	Yes	17	60	D^+^
4	HTS	Female	89	Leg	+	*NA*	-	+	*NA*	RT	Yes	21	32	A^-^
5	HTS	Female	84	Leg	+	+	-	+	IgM, IgD, κ	RT	No	0	2	D^0^
6	HTS	Female	83	Leg	+	+	-	+	IgM, IgD, κ	RT + PCT	Yes	34	38	D^+^
7	HTS	Male	83	Leg	+	+	-	+	IgM, IgD, κ	RT	No	49	50	A^0^
8	HTS	Female	76	Leg	+	-	-	+	IgM, κ	RT	No	0	13	D^+^
9	HTS	Male	77	Both legs	+	-	-	+	IgM, IgD	RT	No	0	3	D^+^
10	HTS	Female	79	Leg	+	+	-	+	IgM, κ	RT	?	>20	32	A^?^
11	HTS	Female	81	Leg	+	-	-	+	IgM	RT + PCT	Yes	20	39	D^+^
12	RT-PCR	Female	73	Leg	+	+	-	+	IgM, IgD, κ	PCT	No	79	85	D^0^
13	RT-PCR	Female	71	Leg	+	-	-	+	IgM, λ	PCT	Yes	34	73	A^0^
14	RT-PCR	Male	47	Leg	+	+	-	+	IgM, IgD, λ	PCT	No	43	49	A^0^
15	RT-PCR	Female	75	Leg	+	+	-	-	κ ^a^	PCT	Yes	2	29	D^+^
16	RT-PCR	Female	77	Leg	+	-	-	+	IgM, λ	PCT	Yes	10	19	D^+^
17	RT-PCR	Female	75	Both legs	+	-	-	-	IgM, IgD, κ	PCT	Yes	1	12	D^+^
18	RT-PCR	Female	84	Leg	+	+	-	+	IgM, IgD, κ	None	No	0	3	D^+^

*NA*: not assessed; RT: radio-therapy; PCT: polychemotherapy; A^+^: alive with disease; A^0^: alive without disease; D^+^: death with disease; D^0^: death without disease; ^a^ heavy chains not assessed. BCR: B-cell receptor; PFS: progression-free survival; HTS: high-throughput sequencing; RT-PCR: Real Time polymerase chain reaction;

Human B-cells were purified from peripheral blood mononuclear cells isolated from buffy coats obtained from anonymous blood donors (Sanquin Bloodbank, Nijmegen, The Netherlands) and were in vitro activated subsequently (details are given in File S1).

### MicroRNA library preparation, high-throughput sequencing and data analysis

Total RNA was extracted from the frozen tumor samples and activated B-cells using TRIzol and according to the protocol described by the supplier (Invitrogen, Carlsbad, CA, USA). Library preparation was to a large extent based on the SOLiD Total RNA-Seq Kit protocol (SREK protocol, Applied Biosystems, Nieuwerkerk a/d IJssel, The Netherlands) as described previously (File S1) [25]. Subsequently, 0.5 nM per sample of the cDNA clusters generated was loaded onto the flow cells for massively parallel high-throughput sequencing using the Illumina Genome Analyzer II (Illumina) performing sequencing-by-synthesis technology. The raw sequencing data were extracted through standard Illumina pipelines. The primary sequencing data and are publicly available through the Gene Expression Omnibus (GEO) archive through accession GSE51359. Illumina sequence reads were aligned to the human genome (GRCh37/hg19) using the miRDeep2.0 algorithm [26], which predicts precursor sequences and matches them to precursor microRNAs enlisted in the miRBase 18.0 database (http://www.mirbase.org). Only sequences with a minimum sequence length of 17 bases were analyzed, allowing for a maximum mismatch of one base. Samples with less than 100.000 microRNA reads were discarded from further analyses [27]. The different microRNAs were only included in the analyses if in at least half of the samples of at least one of the three analyzed groups (PCFCL, PCLBCL-LT or activated B-cells) the single microRNA read number exceeded 0,01% of the total number of microRNA reads (100 reads/million). The samples were then analyzed for differential expression between the groups by the Bioconductor package EdgeR [28], considering only mature microRNAs. Differential expression was considered statistically significant when a *p*-value below 0.05 was reached after multiple testing correction according to the Benjamini-Hochberg method [29]. EdgeR transformed, normalized microRNA expression (in counts per million reads) were used for unsupervised hierarchical clustering, running under R 2.14.2 software (http://www.r-project.org).

The NormFinder version 0.953 algorithm [30] was used on high-throughput sequencing data to identify the most stably expressed microRNAs within and between the analyzed subgroups, suitable for RT-qPCR normalization [31].

### MicroRNA real-time qPCR

Total RNA was isolated from 10 µm sections of FFPE tumour samples. Approximately 300 ng of total RNA from each sample (FFPE or frozen, same isolation as for the high-throughput sequencing) was reverse transcribed using the microRNA reverse transcription kit (Applied Biosystems). RT-qPCR was performed in duplicate using Taqman microRNA and control assays (Applied Biosystems). The output data were analyzed using CFX Manager software (Bio-Rad). The expression of the different microRNAs was analyzed using the ΔΔCt method expressed relative to the most stably expressed endogenous controls according to GeNorm [32]. For more details see File S1. Statistical analyses were performed using the Mann Whitney U test in IBM SPSS version 20 (SPSS Inc., Chicago, IL, USA) and the data were visualized in GraphPad Prism (GraphPad Software Inc, CA, USA).

## Results

### High-throughput sequencing

From 19 tumor samples (13 samples of PCLBCL-LT and 6 samples of PCFCL) and 4 samples of activated B-cells, microRNA libraries were generated and sequenced. Of these, two PCLBCL-LT samples yielded less than 100.000 total microRNA reads (7.302 and 55.383 reads) and were discarded from further analyses [27]. Mean microRNA read number in the other samples was 4.139.515 (standard deviation 9.348.501). After alignment of the sequence reads in miRDeep 2.0, 1921 different known precursor microRNAs were identified to be expressed in at least one sample (Table S2). 328 precursor loci were identified as putative novel microRNAs (Table S3). After discarding precursor microRNAs that showed low expression levels (see Materials and Methods), a total number of 238 different precursor microRNAs were included for further analysis.

### MicroRNA profiles of PCFCL and PCLBCL-LT

**Figure 1:**
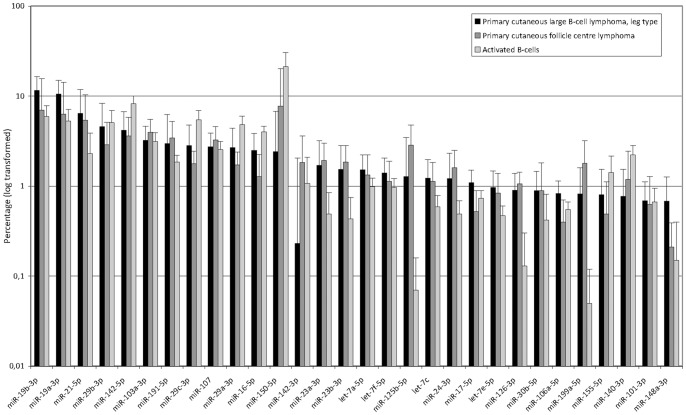
Most abundantly expressed microRNAs. From left to right the mean of the top microRNAs expressed in PCLBCL-LT are listed and compared to the other subgroups.

The most abundantly expressed microRNAs of the analyzed subgroups are represented in Figure 1. In PCLBCL-LT, miR-19b-3p and miR-19a-3p show the highest expression levels, and comprise 12% and 11% of the total microRNome, respectively. In PCFCL, these microRNAs also constitute a substantial part of the microRNome (7% and 6%, respectively), but miR-150-5p is most abundantly expressed. The activated B-cells are to a large extent represented by miR-150-5p, comprising approximately one-fifth of the microRNome. Using NormFinder, miR-148b-3p, miR-25-3p, let-7c and let-7e-5p were the most stably expressed microRNAs in our complete study cohort (Figure S1 in File S2).

Unsupervised hierarchical clustering analysis of all samples using the 238 mature microRNAs of the precursor sequences identified did not result into distinct clustering of the two tumor types and/or the activated B-cells (Figure S2 in File S2).

### Differential microRNA expression in PCLBCLs

**Figure 2:**
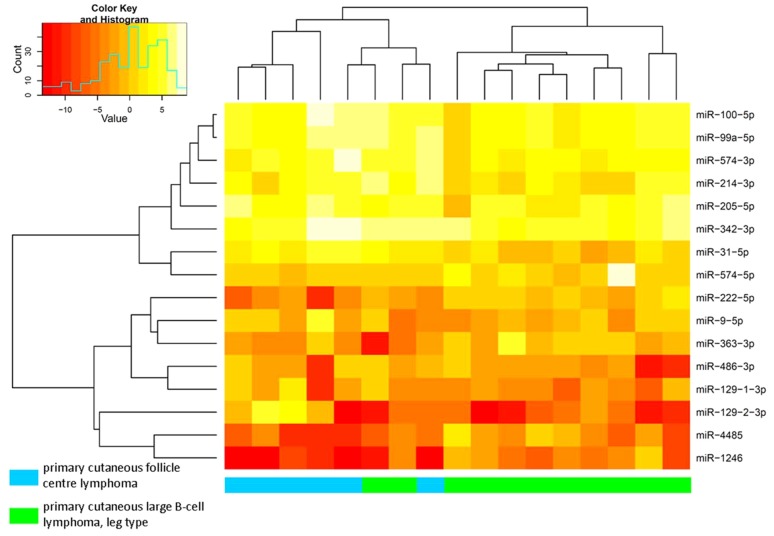
Unsupervised hierarchical clustering analysis in PCLBCL samples of high-throughput sequencing, using 16 differentially expressed microRNAs. PCFCL and PCLBCL-LT tend to cluster together in two subgroups, with only 2 cases of PCLBCL-LT clustering together with the PCFCL cases.

Table 2 shows an overview of mature microRNAs that showed statistically significantly differential expression between PCFCL and PCLBCL-LT. Under the given conditions (allowing a mismatch of 1 base pair), MiRDeep 2.0 did not categorize miR-129-1-3p and miR-129-2-3p as separate microRNAs. Recounting of both variants in the aligned sequence data derived from miRDeep 2.0 showed a markedly higher expression of miR-129-2-3p in PCFCL as compared to PCLBCL-LT. Similarly, miRDeep 2.0 was not able to differentiate between miR-9-1, -2 and -3 variants, because of the relatively short aligned sequences (no sequences containing (part of) the precursor microRNA outside the mature microRNA region). For the 16 differentially expressed microRNAs, unsupervised hierarchical clustering analysis was applied (Figure 2). According to this analysis, two groups are separated from each other, one containing all cases of PCFCL and two cases of PCLBCL-LT, the other group comprising of the remaining cases of PCLBCL-LT. The two cases of PCLBCL-LT clustering with the PCFCL cases showed a clinical presentation, tumor cell morphology and immunohistochemical profile with classical features of PCLBCL-LT. One of the patients received polychemotherapy combined with radiotherapy and died of lymphoma after 38 months; the other patient was solely treated with radiotherapy and is still alive without disease recurrence after 50 months of follow-up.

**Table 2:** High-throughput sequencing: differential expression of microRNAs between PCFCL and PCLBCL-LT.

MicroRNA		Reads per million, mean (*SD*)		Adjusted *p*-value^a^
		PCFCL	PCLBCL-LT	
MiR-129-2-3p	b	1.668 (*3.260*)	4 (*8*)	5.74E-15
MiR-129-1-3p		184 (*269*)	18 (*21*)	2.10E-14
MiR-1246		0 (*0*)	26 (*45*)	1.42E-5
MiR-214-3p	b	8.620 (*14.534*)	1.174 (*1.232*)	7.24E-4
MiR-574-3p	b	12.079 (*14.180*)	1.951 (*1.392*)	0.00266
MiR-4485		6 (*8*)	77 (*127*)	0.00321
MiR-222-5p		16 (*14*)	142 (*126*)	0.0108
MiR-31-5p	b	1.729 (*995*)	346 (*385*)	0.0129
MiR-486-3p	b	196 (*194*)	40 (*43*)	0.0133
MiR-363-3p	b	37 (*27*)	288 (*535*)	0.0153
MiR-99a-5p	b	9.843 (*9.113*)	2.168 (*1.155*)	0.0184
MiR-574-5p		289 (*184*)	1.996 (*5.654*)	0.0442
MiR-100-5p	b	8.849 (*6.850*)	2.318 (*1.400*)	0.0442
MiR-205-5p	b	11.492 (*12.787*)	3.059 (*2.818*)	0.0442
MiR-342-3p	b	22.775 (*24.636*)	6.079 (*3.992*)	0.0442
MiR-9-5p	b	273 (*309*)	73 (*74*)	0.0442

*p*-values Benjamini-Hochberg corrected.^a^

b microRNA included in RT-qPCR validation.

PCFCL: primary cutaneous follicle center lymphoma, PCLBCL-LT: primary cutaneous large B-cell lymphoma, leg type.

### MicroRNA RT-qPCR validation

A selection of 11 differentially expressed microRNAs (marked in Table 3) was used for validation in a novel group of test cases. MiR-129-1-3p, miR-1246, miR-4485 and miR-222-5p were not validated because of the relatively low counts in both PCFCL and PCLBCL-LT samples. Of miR-574-5p, no Taqman PCR primer assay was available, because of dinucleotide repeats in the microRNA sequence that could interfere with the PCR. Assays for miR-148b-3p, miR-25-3p, let-7c and let-7e-5p were included as reference microRNAs, along with widely used reference RNAs U6 and RNU48. Of these six, miR-148b-3p, let-7e-5p and miR-25-3p were most stably expressed in the RT-qPCR experiments (according to GeNorm) and therefore used for normalization of the data. The expression of U6 and RNU48 was very variable in this sample set. Comparison of RT-qPCR performed on both RNA of frozen tumor material isolated for high-throughput sequence analysis and RNA isolated from FFPE sections of the same tumor sample (2 cases of PCFCL and 3 cases of PCLBCL-LT) showed high concordance (Figure S3 in File S2).

**Table 3:** MicroRNAs in RT-qPCR validation: predicted targets.

MicroRNA	Chromosomal location microRNA gene	Up^a^	Potential target(s) and function	
MiR-129-2-3p	11p11.2	PCFCL	SOX4	(B-cell) transcription factor
			CDK6	Regulator of cell cycle progression
MiR-214-3p	1q24.3	PCFCL	PTEN	Negative regulator of cell cycle progression
			EZH2	Polycomb group gene silencer
MiR-574-3p	4p14	PCFCL	IL6	Involved in NF-κB signaling
MiR-31-5p	9p21.3	PCFCL	PIK3C2A	PI3K pathway component
			CXCL12	Chemokine attractant
			RhoA	Metastasis promoting gene
			NIK	NF-κB signaling
MiR-486-3p	8p11.21	PCFCL	PTEN	Negative regulator of cell cycle progression
			E2F1	Regulator of cell cycle progression
MiR-99a-5p	21q21.1	PCFCL	mTOR	PI3K family member
MiR-100-5p	11q24.1	PCFCL	mTOR	PI3K family member
MiR-205-5p	1q32.2	PCFCL	PTEN	Negative regulator of cell cycle progression
			E2F1	Regulator of cell cycle progression
MiR-342-3p	14q32.2	PCFCL	BCL2L10	BCL2 family member
MiR-9-5p	1q22/5q14.3/15q26.1	PCFCL	PRDM1/BLIMP1	B-cell transcription factor
			FOXP1	(B-cell) transcription factor
			NFκB1 = p50	NF-κB signaling
MiR-363-3p	Xq26.2	PCLBCL-LT	CDKN1A = p21	Regulator of cell cycle progression

a higher expression in subgroup of PCLBCL.

PCFCL: primary cutaneous follicle center lymphoma; PCLBCL-LT: primary cutaneous diffuse large B-cell lymphoma, leg type.

**Figure 3:**
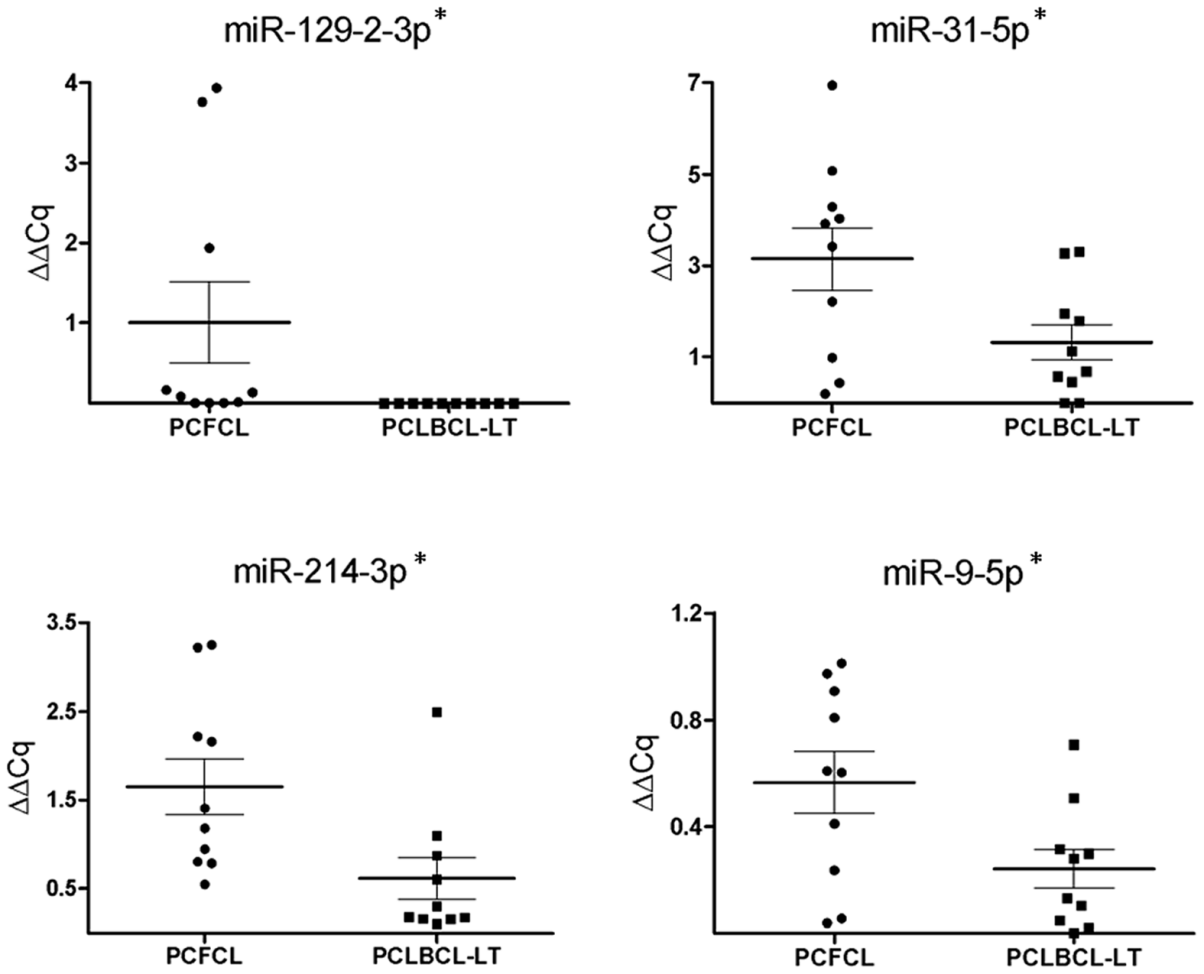
RT-qPCR expression in FFPE samples (1). The expression of the 4 microRNAs showing statistically significantly higher expression in PCFCL as compared to PCLBCL-LT are represented in dot plots. ΔΔCq was calculated with miR-148b-3p, let-7e-5p and miR-25-3p as stably expressed reference microRNAs. *P-value < 0.05 (Mann Whitney U test).

**Figure 4:**
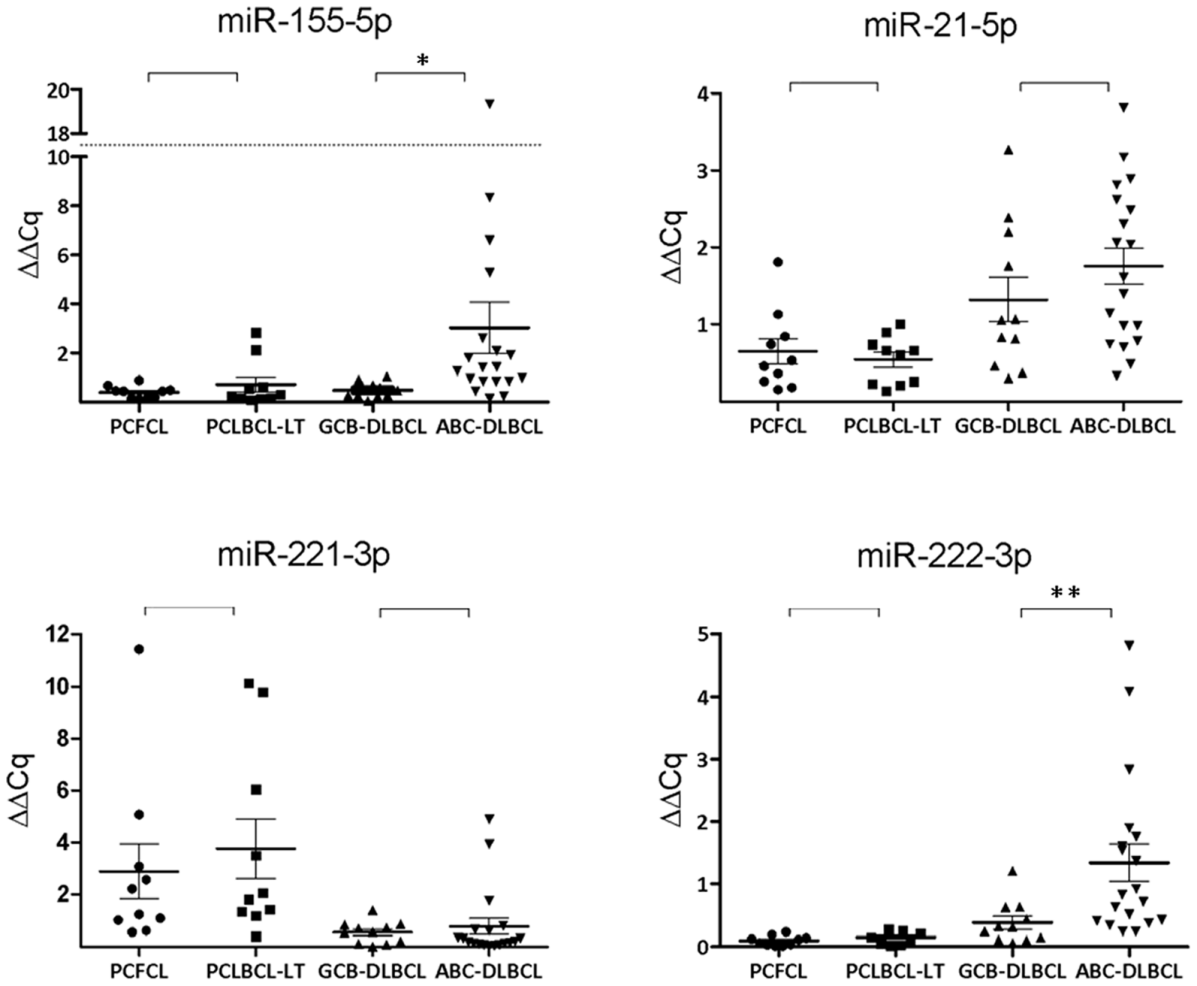
RT-qPCR expression in FFPE samples (2). The expression of the 4 microRNAs that are repeatedly reported in previous studies to be more abundantly expressed in ABC-type than GCB-type nodal DLBCL are represented in dot plots. ΔΔCq was calculated with miR-148b-3p, let-7e-5p and miR-25-3p as stably expressed reference microRNAs. *P-value < 0.05; ** P-value  =  0.05 (Mann Whitney U test).

With inclusion of all 20 FFPE PCLBCL samples in the analysis, including 10 PCFCL and 10 PCLBCL-LT cases, we confirmed higher expression of miR-129-2-3p, miR-214-3p, miR-31-5p and miR-9-5p in PCFCL as compared to PCLBCL-LT (*p*<0.05) (Figure 3). For the other seven microRNAs, no statistically significantly differential expression was found between PCFCL and PCLBCL-LT. In addition to these differentially expressed microRNAs resulting from the deep sequence analysis, we tested whether four microRNAs frequently reported to show higher expression in ABC-type compared GCB-type nodal DLBCL (miR-21-5p, miR-155-5p, miR-221-3p and miR-222-3p) were higher expressed in PCLBCL-LT. However, no differential expression between PCLBCL-LT and PCFCL of these four microRNAs was observed (Figure 4). Above that, unsupervised hierarchical clustering analysis of the high-throughput sequence data of our PCLBCLs using microRNA signature profiles that should be able to distinguish GCB-type from ABC-type DLBCL [12], [14], [17] did not set apart PCFCL from PCLBCL-LT.

### MicroRNA RT-qPCR analysis of GCB- and ABC-subtypes of nodal DLBCLs

Because of the inconsistencies in literature concerning microRNA profiling of nodal DLBCL and the varying references used in these studies, a set of well-defined GCB- and ABC-subtype nodal DLBCLs was subjected to microRNA RT-qPCR analysis, using the same conditions as for our PCLBCL FFPE samples. As in PCLBCL, GeNorm analysis showed that miR-148b-3p, miR-25-3p, let-7e-5p were most stably expressed, and these three microRNAs were therefore used as reference microRNAs. We evaluated the expression of the four microRNAs that showed significantly higher expression in PCFCL as compared to PCLBCL-LT in our nodal DLBCL samples. The expression of miR-129-2-3p was indeed higher in GCB-type DLBCL as compared to the ABC-type (*p* = 0.00), while the expression of miR-214-3p, miR-31-5p and miR-9-5p did not show statistically significant differences between both nodal subtypes. Four microRNAs frequently reported to show higher expression in ABC-type than in GCB-type nodal DLBCL (miR-21-5p, miR-155-5p, miR-221-3p and miR-222-3p) were included in the RT-qPCR analysis. However, in our group of nodal DLBCLs, of these four microRNAs, we could only confirm higher expression levels of miR-155-5p (*p* = 0.01) and miR-222-5p (*p* = 0.05) in ABC-type as compared to GCB-type DLBCL (Figure 4).

## Discussion

This is the first study to report complete genome-wide microRNA profiles of PCLBCLs, and comparing PCFCL and PCLBCL-LT subtypes. By use of next generation high-throughput microRNA sequence analysis, we provided microRNA profiles of these tumors on frozen biopsy samples, accurately exploring both quantitative and qualitative aspects of all microRNAs. The complete microRNomes of PCLBCL-LT and PCFCL did not allow their separation by unsupervised hierarchical clustering, and PCLBCL-LT did not cluster together with activated B-cells, its postulated normal counterpart (Figure S1 in File S2). The list of expressed and quantified microRNAs generated from this experiment was the basis for selecting specific microRNAs for RT-qPCR confirmation on FFPE tumor tissue of both lymphoma subtypes. The use of high-throughput sequencing also enabled us to identify 328 putative novel microRNAs expressed in our samples, of which further validation was not performed and is warranted. Furthermore, these sequence data were also instrumental in selecting stably expressed reference microRNAs for subsequent confirmation experiments. We identified four microRNAs (miR-129-2-3p, miR-214-3p, miR-31-5p and miR-9-5p) of which statistically significantly higher expression in PCFCL as compared to PCLBCL-LT in high-throughput sequencing could be confirmed by RT-qPCR on a new FFPE study cohort.

### MicroRNomes of PCFCL and PCLBCL-LT

Considering all samples analyzed, miR-19a-3p and miR-19b-3p constituted a significant part of the microRNA profile, up to 12% for miR-19b-3p of the complete profile of PCLBCL-LT. Both microRNAs are members of the miR-17∼92 cluster, the presence of which is critical for normal B-cell development [33]. It also acts as a potential oncogenic cluster in B-cell non-Hodgkin lymphomas [34], with miR-19 components being essential in this oncogenic potential [35]. The other members of this cluster, miR-17-5p, miR-18a-5p, miR-20a-5p and miR-92a-3p, only made up a very small proportion of the complete microRNome (with a maximum of 1%). The relatively low expression of miR-92a-3p was most remarkable, as this microRNA was found to be one of the most abundantly expressed microRNA in a recent study on nodal DLBCL and primary central nervous system DLBCL, an extranodal DLBCL with an ABC-like genotype [36]. Although in PCFCL, miR-19a-3p and miR-19b-3p also made up a substantial part of the microRNome, miR-150-5p was most abundantly expressed. MiR-150-5p is involved in normal B-cell development, targeting transcription factor and proto-oncogene c-MYB [37].

### Differentially expressed microRNAs

By performing RT-qPCR in an independent cohort of FFPE tumor samples, the higher expression of miR-129-2-3p, miR-214-3p, miR-31-5p and miR-9-5p in PCFCL as compared to PCLBCL-LT in high-throughput sequence data could be confirmed.

MiR-129-2-3p targets transcription factor SOX4, which was validated in endometrial cancer [38]. SOX4 also has a critical function in normal B-cell development [39]. Because of the dichotomy in expression of miR-129-2-3p (11 cases without and 9 cases with expression of this microRNA in RT-qPCR), immunohistochemical staining for SOX4 was performed. However, no inverse correlation between RT-qPCR expression of miR-129-2-3p and immunohistochemical expression of SOX4 was observed (data not shown).

A previous array-based comparative genomic hybridization study performed by our group, showed low copy number gains of 1q23-q25 in PCFCL, but not in PCLBCL-LT cases [5]. The miR-214 gene is located in this chromosomal region and correspondingly, higher levels of miR-214-3p were observed in PCFCL compared to PCLBCL-LT. MiR-214-3p has been reported as oncogenic microRNA in T-cells, negatively regulating tumor suppressor gene phosphate and tensin homolog (PTEN) [40], and is involved in controlling cell cycle regulation. However, miR-214-3p has also been described to exert a tumor suppressor function in breast cancer cell lines, where reduced levels of this microRNA result in increased proliferation and invasion and in accumulation of EZH2 [41], a polycomb group protein with the potential to promote cell proliferation [42].

The miR-31 gene is located on 9p21.3. This chromosomal region is often targeted by monoallelic or biallelic deletions in PCLBCL-LT, but not in PCFCL [4], [43]. In accordance, the expression of miR-31-5p was lower in PCLBCL-LT in proportion to PCFCL. Correlation of previous multiplex ligation probe-dependent assay results of chromosomal location 9p21.3 [43] to the current results showed that two of the PCLBCL-LT samples tested here, had deletions in this region and low expression of miR-31-5p, while four samples did not have deletions and showed higher expression of this specific microRNA. Well characterized genes involved in this deletion are CDKN2A and CDKN2B, tumor suppressor genes involved in cell cycle control [44]. We previously reported that inactivation of CDKN2A in PCLBCL-LT is associated with inferior survival [4], [43]. It is however not clear whether the miR-31 gene is also part of the 9p21.3 deletion involved in this more aggressive behavior. Downregulation of miR-31-5p has been described in different types of T-cell non-Hodgkin lymphomas [45]. In these lymphomas, miR-31-5p was shown to be associated with activation of the nuclear factor (NF)-κB pathway, a signaling pathway often deregulated in several types of malignancies, by targeting NF-κB inducing kinase [45].

MiR-9-5p is involved in normal B-cell development, negatively regulating transcription factor PRDM1, an essential driver of plasma cell differentiation. In accordance, this microRNA is more abundantly expressed in normal germinal center B-cells than plasma cells [21]. As PCLBCL-LT is thought to be derived from B-cells in a stage just beyond the germinal center reaction, tending towards plasma cell differentiation, the lower expression of miR-9-5p might reflect a normal biological stage in B-cell maturation. In ovarian cancer, miR-9-5p has been demonstrated to act as a tumor suppressor microRNA by targeting the NF-κB family member NFκB1/p50 [46]. Since the NF-κB pathway has a well-known oncogenic potential in ABC-type nodal DLBCL [47], it can be speculated that downregulation of miR-9-5p might have a pathogenic effect in PCLBCL-LT through reduced inhibition of NF-κB signaling.

### Correlation with nodal DLBCL subtypes

PCFCL and PCLBCL-LT have a very distinct gene expression profile, corresponding with gene expression profiles of GCB- and ABC-type nodal DLBCL [5]. We therefore were interested in comparing both subtypes of PCLBCL with their molecular counterparts of nodal DLBCL. However, literature concerning microRNA expression in nodal DLBCL shows inconsistent results. Taken together, the most frequently reported microRNAs showing higher expression in ABC-type as compared to GCB-type nodal DLBCL are miR-155-5p, miR-21-5p and miR-221-3p and miR-222-3p [11], [12], [15]-[19], [48]. In our PCLBCL samples, expression of these four microRNAs did not show significant differences between the two subtypes in both high-throughput sequencing and RT-qPCR. To accurately compare our PCLBCL samples with nodal DLBCL, and exclude external influences such as differing reference microRNAs, we performed RT-qPCR on well-defined subgroups of nodal DLBCL by using a set of three microRNAs (miR-148b-3p, let-7e-5p and miR-25-3p) that were stably expressed in our PCLBCL sequencing studies as a reference. These microRNAs were, in contrast to widely used references U6 and RNU48, stably expressed in our nodal DLBCLs. By using this approach of matching conditions, we confirmed higher expression of miR-155-5p and miR-222-3p in ABC-type as compared to GCB-type nodal DLBCL, but not of miR-21-5p and miR-221-3p.

The absence of higher expression of miR-155-5p in PCLBCL-LT as compared to PCFCL was most noteworthy. The role miR-155-5p has been studied extensively in nodal B-cell non-Hodgkin lymphomas and is linked to activation of the intracellular signaling pathway PI3K-Akt pathway [10] and increased lymphoma cell motility [49], and its expression is associated with NF-κB activity in nodal DLBCL [48]. The finding that miR-155-5p was not elevated in PCLBCL-LT as compared to PCFCL suggests at least partially different pathogenetic mechanisms in PCLBCL-LT as compared to its nodal molecular counterpart.

In summary, we presented the first microRNA profiling results by next generation high-throughput sequencing and RT-qPCR comparing PCFCL and PCLBCL-LT. These two subtypes of PCLBCL did not show entirely distinct microRNA profiles, but could be distinguished by differential expression of microRNAs miR-129-2-3p, miR-214-3p, miR-31-5p and miR-9-5p. We also showed that the hitherto described microRNA signature of GCB- and ABC-subtype nodal DLBCLs could not be extrapolated to PCFCL and PCLBCL-LT. Therefore, we conclude that although PCFCL and PCLBCL-LT show strong resemblance at gene expression level with GCB- and ABC-type nodal DLBCL, respectively, their microRNA profiles are at least partially disparate from their nodal counterparts, suggesting distinct pathogenetic mechanisms.

## Supporting Information

File S1 **Supplementary Materials and Methods.**
(DOC)

File S2 **Figure S1.** Most stably expressed microRNAs in high-throughput sequencing according to GeNorm. **Figure S2.** Unsupervised hierarchical clustering of the normalized expression of all analyzed microRNAs of high-throughput sequencing. **Figure S3.** Internal validation.
(PDF)

Table S1 **Complete microRNA profiling of nodal DLBCL: differences between ABC- and GCB-type nodal DLBCL.**
(PDF)

Table S2 **Raw data high-throughput sequencing.**
(XLS)

Table S3 **Putative novel microRNAs.**
(PDF)
