# Supplementary material for: MicroRNA Profiling of Primary Cutaneous Large B-Cell Lymphomas
Source: PLoS One. 2013 Dec 16;8(12):e82471. doi: 10.1371/journal.pone.0082471 (PMC3865085; doi:10.1371/journal.pone.0082471)
Supplement: File S1 — Supplementary Materials and Methods. (DOC) [file pone.0082471.s001.doc]

**File S1. Supplementary Materials and Methods**

**B-cell selection and activation**

Human B-cells were purified from the separate lymphocyte fractions of peripheral blood (buffy coats) obtained from four healthy individuals by positive selection with magnetic CD19 MicroBeads (Miltenyi Biotec, Bergisch Gladbach, Germany). The B-cells were cultured in RPMI 1640 with 10% FCS and incubated with CpG (5 μg/ml) (ODN 2006, Invivogen, San Diego, USA) and monoclonal antibodies to CD40 (0.1 μg/ml) (BD Biosciences Pharmingen, Heidelberg, Germany) for 24 hours. After harvesting, a B-cell purity of more than 97% was confirmed by flow cytometric analysis with a mixture of monoclonal antibodies against B-cell (CD20, APC-H7), T-cell (CD3, PE-H7) and monocytes (CD14, APC). Simultaneously, the activation status was determined using anti-CD80 (PE) and anti-CD86 (FITC)), respectively. All antibodies were obtained from BD Biosciences. Dead cells were excluded using 1 µM of DAPI. A BD Biosciences LSRII was used for acquisition. WinList 7 (Verity Software House, Topsham, ME, USA) was used for data analysis. Approximately 86% of the lymphocytes showed expression of these activation markers.

**MicroRNA library preparation for high-throughput sequencing**

Sequencing adaptors were ligated to total RNA on both ends of the RNA molecules and reverse-transcribed. The cDNA was pre-amplified with PCR primers containing specific sequence tags making the libraries compatible with the Illumina flow cells (Illumina, Son en Breugel, The Netherlands). Polyacrylamide gel electrophoresis size selection was performed, excising the 95-105 bp band containing the adaptor-ligated microRNA insert. Quantification and size verification was performed using a high sensitivity DNA chip (Agilent, Amstelveen, The Netherlands).

**MicroRNA real-time qPCR**

Total RNA was isolated from 10 μm sections of FFPE tumour samples (number of sections varying from 4 to 20 sections, according to the size of the specimen) using the RecoverAll Total Nucleic Acid Extraction Kit according to manufacturer’s protocol (Ambion, Warrington, UK). Approximately 300 ng of total RNA from each sample (FFPE or frozen, same isolation as for the high-throughput sequencing) was reverse transcribed using the microRNA reverse transcription kit (Applied Biosystems) combined with the stem-loop Megaplex primer pool A v2.1 (Applied Biosystems), which allowed for simultaneous reverse transcription of 377 microRNAs and 4 endogenous controls. The reverse transcription was conducted on the LightCycler480 (Roche, Almere, The Netherlands), running 40 cycles with the following conditions: 2 minutes at 16^o^C, 1 minutes at 42^o^C and 1 second at 50^o^C, followed by 5 minutes holding at 85^o^C. RT-qPCR was performed using Taqman microRNA and control assays and 2x Universal PCR mastermix (Applied Biosystems). The reactions were run in duplicate on the CFX384 RT-qPCR Detection System, according to manufacturer’s protocol (Bio-Rad Laboratories, Veenendaal, the Netherlands), with the following cycle parameters: 10 min at 95 ^o^C, followed by 50 cycles denaturing for 15 s at 95 ^o^C and annealing and extending for 60 s at 60 ^o^C.
