# Supplementary material for: MicroRNA Profiling of Primary Cutaneous Large B-Cell Lymphomas
Source: PLoS One. 2013 Dec 16;8(12):e82471. doi: 10.1371/journal.pone.0082471 (PMC3865085; doi:10.1371/journal.pone.0082471)
Supplement: File S2 — Figure S1. Most stably expressed microRNAs in high-throughput sequencing according to GeNorm. Figure S2. Unsupervised hierarchical clustering of the normalized expression of all analyzed microRNAs of high-throughput sequencing. Figure S3. Internal validation. (PDF) [file pone.0082471.s002.pdf]

Figure S1

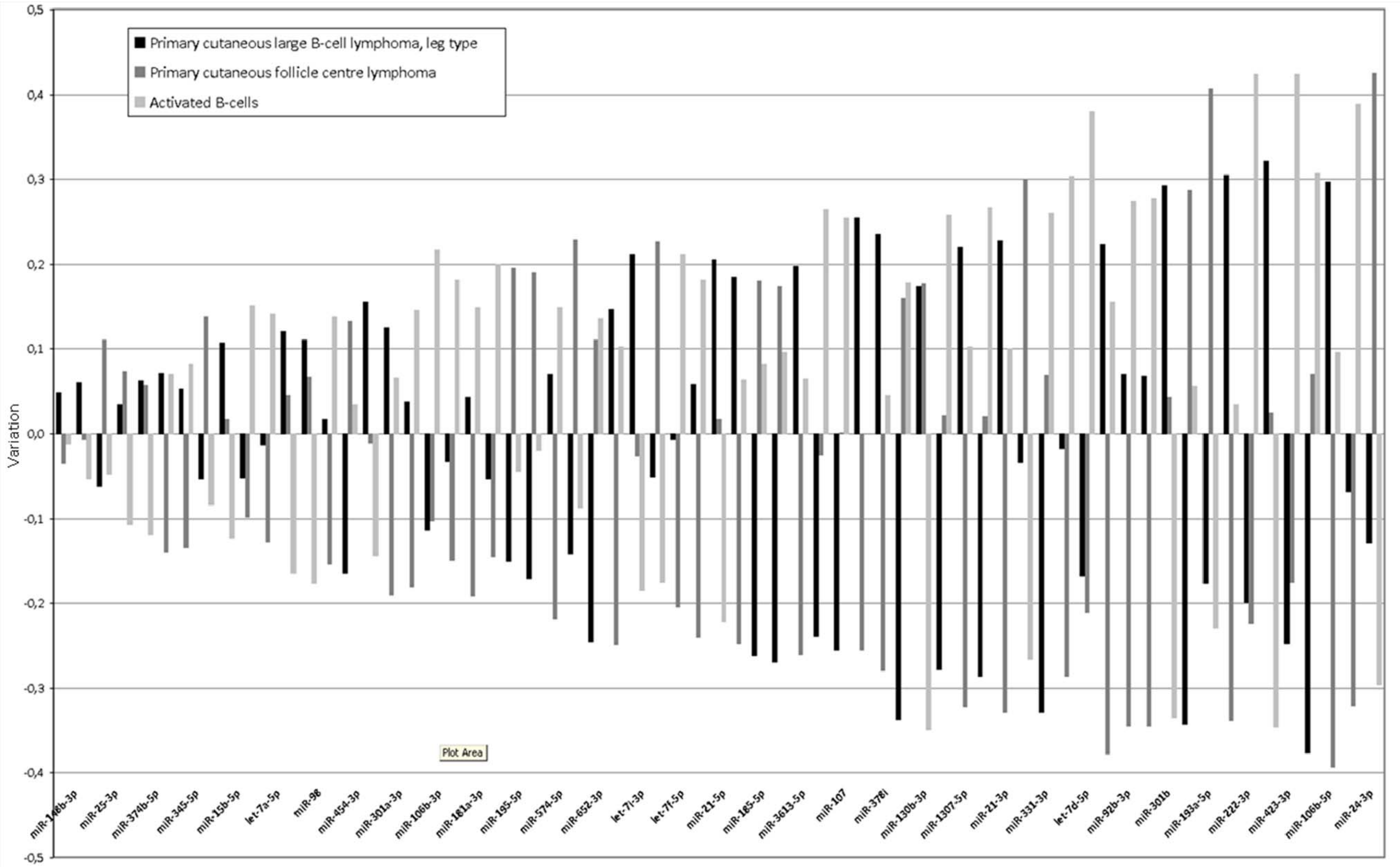

Most stably expressed microRNAs (n=65) in high-throughput sequencing according to GeNorm orderly depicted from left to right. The variation of the three analyzed subgroups per microRNA is plotted on the Y-axis.

Figure S2

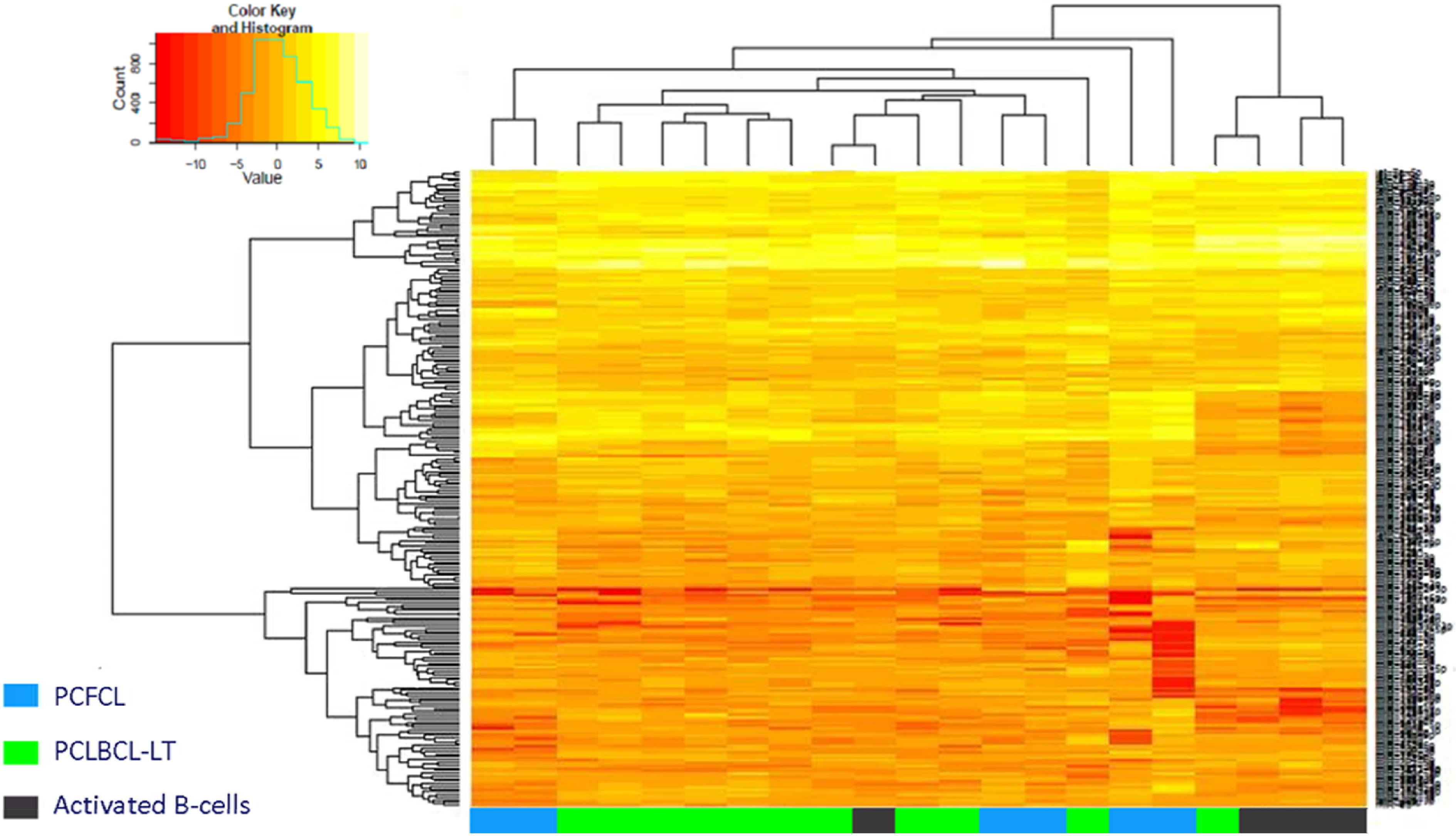

Unsupervised hierarchical clustering of the normalized expression of all 238 analyzed microRNAs of high-throughput sequencing for PCFCL, PCLBCL-LT and activated B-cell samples. There is no evident clustering of the analyzed subgroups.

Figure S3

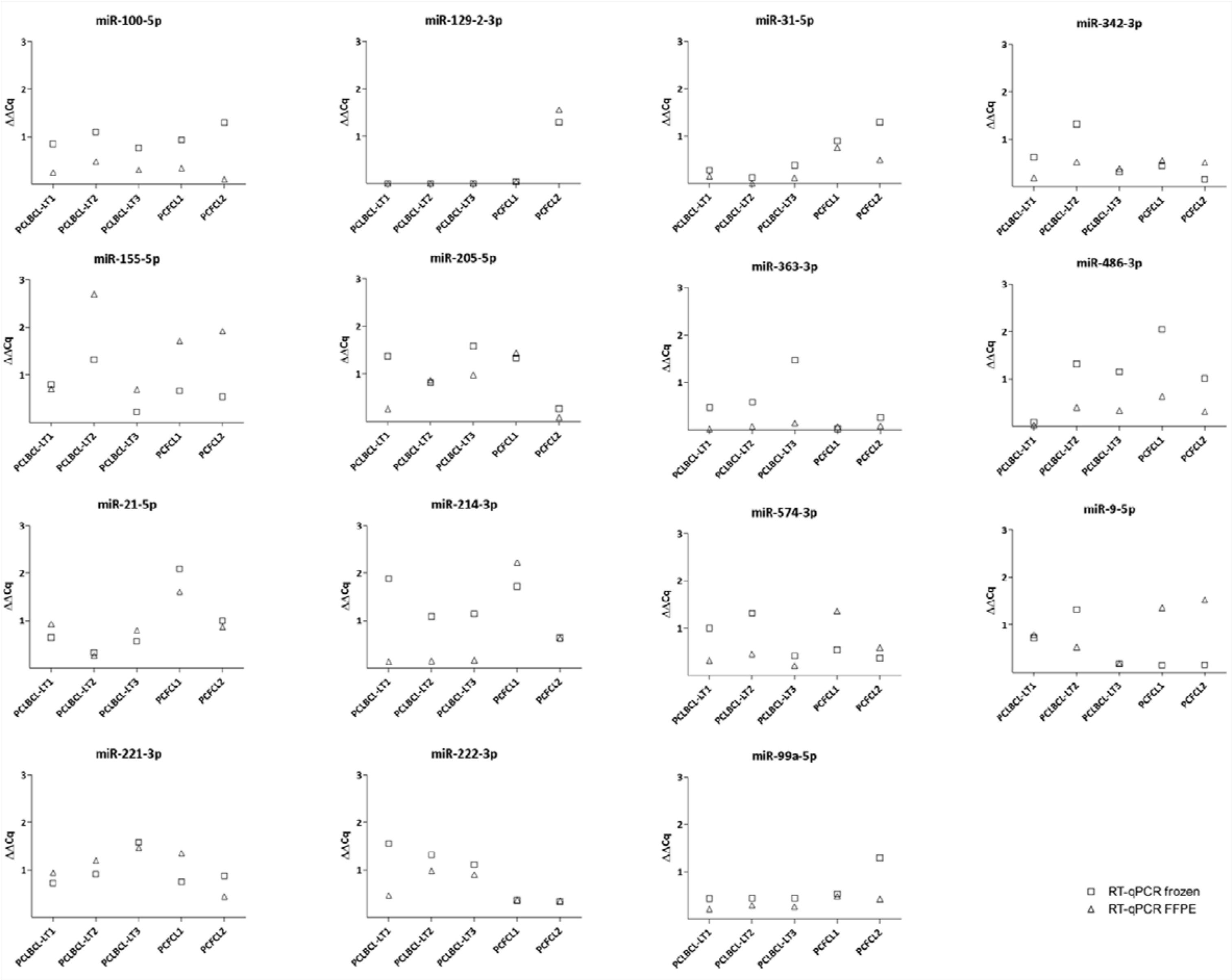

Internal validation. Graphical representation of RT-qPCR data of frozen tumour samples (same RNA as used for high-throughput sequencing) and FFPE tumour samples (same tumour as frozen sample). ΔΔCq values are calculated relative to miR-148b-3p, let-7e-5p and miR-25-3p.
