# Supplementary material for: MicroRNA Profiling of Primary Cutaneous Large B-Cell Lymphomas
Source: PLoS One. 2013 Dec 16;8(12):e82471. doi: 10.1371/journal.pone.0082471 (PMC3865085; doi:10.1371/journal.pone.0082471)
Supplement: Table S1 — Complete microRNA profiling of nodal DLBCL: differences between ABC- and GCB-type nodal DLBCL. (PDF) [file pone.0082471.s003.pdf]

Table S1. Complete microRNA profiling of nodal DLBCL: differences between ABC- and GCB-type nodal DLBCL

Author	Journal	Year	# pt	Technique	MicroRNA ↑ in ABC- vs GCB-DLBCL	MicroRNA ↑ in GCB- vs ABC-DLBCL	Subtyping DLBCLs
Lawrie	Int J Cancer	2007	5*	miRMAX microarray (252 microRNAs)	**miR-155**, **miR-21**, **miR-222**	none	established cell lines
Roehle	Br J Haematol	2008	58	qPCR assay (157 microRNAs)	**miR-155**	miR-27b, miR-28, miR-129, miR-133a, miR-133b, miR-138, mir-151	IHC: Hans
Lawrie†	J Cell Mol Med	2009	64	custom-made microarray (464 microRNAs)	miR-518a, miR-363, **miR-21**, miR-132, miR-340, miR-301, miR-30d, **miR-221**, miR-422b, miR-146b, **miR-155**, miR-190, miR-194, miR-660, miR-213, **miR-222**, miR-186	miR-620, miR-616, miR-199b, miR-421, miR-569, miR-653, miR-138, miR-520h, miR-129	IHC: Hans
Malumbres†	Blood	2009	8*	LC Sciences microarray (711 microRNAs)	miR-146b-5p, miR-146a, **miR-21**, **miR-155**, miR-500, **miR-222**, miR-363, miR-574-5p, miR-574-3p	none	established cell lines
Zhang	Blood	2009	40	Exiqon microarray (789 microRNAs)	(*up- or downregulation not stated*) miR-142-3p, miR-16, miR-184, miR-191, miR-19a, miR-19b, miR-299-5p, miR-32, miR-30e*, miR-151-5p, miR-583, mghv-miR-M1-7-5p, miR-142-5p, miR-106b, miR-30e, miR-140-3p, miR-20a, miR-526b*, miR-28-5p, miR-30c		IHC: Hans
Culpin	Int J Oncol	2010	8*	Miltenyi Biotec microarray (1860 microRNAs)	miR-17, miR-19b, miR-20a, miR-29a, miR-92a, miR-106a, miR-720, miR-1260, miR-1280	none	IHC: Hans
Montes-Moreno	Blood	2011	29	Agilent microarray (470 microRNAs)	**miR-222**, miR-144, miR-451, **miR-221**	miR-331, miR-151, miR-28, miR-454-3p	IHC: Choi

* cell lines

† study generated a distinct microRNA subset separating ABC- from GCB-type DLBCL by unsupervised hierarchical clustering analysis

# pt: number of patients; IHC: immunohistochemistry; ABC: activated B-cell; GCB: germinal centre B-cell; DLBCL: diffuse large B-cell lymphoma; qPCR: quantitative polymerase chain reaction; NGS: next generation sequencing
