# Supplementary material for: MicroRNA Profiling of Primary Cutaneous Large B-Cell Lymphomas
Source: PLoS One. 2013 Dec 16;8(12):e82471. doi: 10.1371/journal.pone.0082471 (PMC3865085; doi:10.1371/journal.pone.0082471)
Supplement: Table S3 — Putative novel microRNAs. (PDF) [file pone.0082471.s005.pdf]

Table S3. Putative novel microRNAs

chrom.	start	end	miRDeep2 score	estimated probability	total count	mature count	loop count	star count	significant randfold p-	consensus mature sequence	consensus star sequence	consensus precursor sequence
chr17	30306983	30307058	14343,7	59 +/- 16%	28158	28115	0	43	no	cucccacugcuucacuugacua	agagagagagagagagagagagaga	cucccacugcuucacuugacuaacccuaaaaaauauauauauauguauagagagagagagagagagagagaga
chr11	57278032	57278113	2972,6	12 +/- 19%	5833	4460	0	1373	yes	ggcuggucccagugcagugguguu	cucccacugcuucacuuggcuagu	ggcuggucccagugcaguggugguuuacaccuauugaucaaaaccaguuccuuuuccacucccacugcuucacuuggcuagu
chr15	83424762	83424821	1866,9	59 +/- 16%	3662	3494	0	168	yes	agaccuacuuaucuaccaaca	agguagauagaacaggucuug	agguagauagaacaggucuuguuugcaaaauaaauucaagaccuacuuaucuaccaaca
chr17	19093428	19093483	1260,5	59 +/- 31%	2479	2478	0	1	no	ccacgaggaagagagguagcg	ugaagccggcuuucuggcgu	ccacgaggaagagagguagcguuuucuccugaacgugaagccggcuuucuggcgu
chr11	2985065	2985123	654,5	29 +/- 25%	1290	1289	0	1	no	gagcacuguucguaacccguu	uggguuccauuccggugcaa	gagcacuguucguaacccguuagccuggcuguagcuaauggguuccauuccggugcaa
chr2	70313974	70314029	640,3	77 +/- 6%	1252	1243	0	9	yes	accggccgccgccgggg	cggcaggcggccguga	cggcaggcggccgugagugggguuucggucuuucugcccaccggccgccgccgggg
chr7	129594608	129594677	552,5	42 +/- 16%	1100	1099	0	1	no	cccacugcuucacuugacuagc	uggccgggugcgguggcuca	cccacugcuucacuugacuagcuuuaaaaaacaauaaaaauaaauugcauggccgggugcgguggcuca
chr10	21823749	21823829	516,2	8 +/- 16%	1009	994	0	15	yes	cccgccggggcgcgccggc	ccggcgcccccgccccgu	cccgccggggcgcgccggccugcgcccaacggucaccgccgcccccaccugggccgcaaccgccggcgcccccgccccgu
chr5	178772195	178772246	393,6	48 +/- 23%	776	704	0	72	no	gcggcggcggcggcgg	ggggucggcggcggcggcga	ggggucggcggcggcggcgagccuggcguucgcgggcggcggcggcggcgg
chr7	2671572	2671618	310,4	48 +/- 23%	613	612	0	1	no	gcggcggcggcggcgg	gcgggcgcgggcggcc	gcggcggcggcggcggcgaacaaagaggcggcgggcgcgggcggcc
chr9	97904681	97904745	309,6	42 +/- 16%	615	610	0	5	no	cgggugcuguaggcuuu	ggcacacaccuguaauc	cgggugcuguaggcuuuaaaaaaauaaaaauaaaaagacaggcacaguggcacacaccuguaauc
chr15	83424761	83424821	305,3	30 +/- 23%	599	239	1	359	yes	agaccuacuuaucuaccaaca	aagguagauagaacaggucuug	aagguagauagaacaggucuuguuugcaaaauaaauucaagaccuacuuaucuaccaaca
chrX	101186812	101186874	289,7	58 +/- 8%	564	539	0	25	yes	ggcggcggcggcggcggcggcgg	uccuccgccaccuccgccccug	ggcggcggcggcggcggcggcggcaguaauuguuccugccuccuccgccaccuccgccccug
chrX	49126525	49126566	286,8	48 +/- 23%	567	562	0	5	no	gcggcggcggcggcgg	auggcggcggcggccg	auggcggcggcggccgggcaagauggcggcggcggcggcgg
chr19	45844809	45844856	286,5	77 +/- 6%	567	560	0	7	no	gcgcgcgcguguggugg	guuggugugugugugu	guuggugugugugugugugugugugugugcgcgcgcgcguguggugg
chr7	129074243	129074339	260,5	42 +/- 16%	507	480	24	3	yes	cccgccgcgccuggga	cccggcgcgcggcggu	cccggcgcgcggcgguagggucgccuccggcaaagcgagcugaacccugaggggagccgcugaccagcagcauggaggaccccgccgcgccuggga
chr10	12391561	12391602	259,1	42 +/- 16%	514	513	0	1	no	cggcgcgcggcggcgg	ggcuggcaggcggcggca	ggcuggcaggcggcggcaaaggagccggcgcgcggcggcgg
chr8	80680527	80680566	234,8	41 +/- 23%	464	462	0	2	no	gcggcggcggcggcgg	cggcgacggcggcggccgcgcug	cggcgacggcggcggccgcgcuggcggcggcggcggcgg
chr16	15019798	15019860	233	58 +/- 8%	461	458	0	3	no	ccucaccaucccuucugccugca	caggcagaaguggggcugacagg	caggcagaaguggggcugacagggcagaggguugcgcccccucaccaucccuucugccugca
chr16	16418449	16418511	233	58 +/- 8%	461	458	0	3	no	ccucaccaucccuucugccugca	caggcagaaguggggcugacagg	caggcagaaguggggcugacagggcagaggguugcgcccccucaccaucccuucugccugca
chr16	16462737	16462799	233	58 +/- 8%	461	458	0	3	no	ccucaccaucccuucugccugca	caggcagaaguggggcugacagg	caggcagaaguggggcugacagggcagaggguugcgcccccucaccaucccuucugccugca
chr16	18437870	18437932	233	58 +/- 8%	461	458	0	3	no	ccucaccaucccuucugccugca	caggcagaaguggggcugacagg	caggcagaaguggggcugacagggcagaggguugcgcccccucaccaucccuucugccugca
chr2	29136530	29136616	228,4	48 +/- 23%	456	450	1	5	no	gugcugugaugaugccuu	ggcugugucugagcacc	gugcugugaugaugccuuaauauuugugguuucgacucacugagaguaaaaugaggaccuacaauuccuuggcugugucugagcacc
chr2	29136530	29136616	214,7	41 +/- 23%	429	406	0	23	no	gugcugugaugaugccuu	ggcugugucugagcacc	gugcugugaugaugccuuaauauuugugguuucgacucacugagaguaaaaugaggaccuacaauuccuuggcugugucugagcacc
chr14	103804182	103804243	206	58 +/- 8%	406	176	0	230	yes	gacacaauuugagcuugcuaua	uugcaagcaacacucuguggcagauga	uugcaagcaacacucuguggcagaugaucaaaacugucugacacaauuugagcuugcuaua
chr5	10337469	10337529	200,7	41 +/- 23%	407	400	0	7	no	cuucucaccacugcacuugacua	gucugugugugugugucu	cuucucaccacugcacuugacuaguuuuaagauaaauaaaaugucugugugugugugucu
chr16	85496410	85496471	189,7	77 +/- 6%	375	373	0	2	no	ccgccccccggccccgcgcc	gggaggggccggggcg	gggaggggccggggcgccugggggccccgcagccgcucacgccgccccccggccccgcgcc
chr1	213124819	213124852	176,9	48 +/- 23%	351	342	0	9	no	gcggcggcggcggcgg	ggcgcgcggcggcggc	ggcgcgcggcggcggcagcggcggcggcggcgg
chr15	83424761	83424821	162	42 +/- 16%	318	184	0	134	yes	agaccuacuuaucuaccaaca	aagguagauagaacaggucu	aagguagauagaacaggucuuguuugcaaaauaaauucaagaccuacuuaucuaccaaca
chr22	39901180	39901225	160,4	51 +/- 35%	311	308	0	3	yes	agcgagaccccgucucuu	gagacggagugucacucu	agcgagaccccgucucuuuuuuuuuuggagacggagugucacucu
chr15	83424761	83424821	159,5	41 +/- 31%	313	307	0	6	yes	agaccuacuuaucuaccaaca	aagguagauagaacaggucuugu	aagguagauagaacaggucuuguuugcaaaauaaauucaagaccuacuuaucuaccaaca
chr16	2156680	2156741	147	58 +/- 8%	292	289	0	3	no	ccucaccaccccuucugccugc	caggcagaaguggggcugacagg	caggcagaaguggggcugacagggcagaggguugcgcccccucaccaccccuucugccugc
chr16	15227933	15227994	147	58 +/- 8%	292	289	0	3	no	ccucaccaccccuucugccugc	caggcagaaguggggcugacagg	caggcagaaguggggcugacagggcagaggguugcgcccccucaccaccccuucugccugc
chr5	178772195	178772246	144,9	51 +/- 35%	288	237	0	51	no	gcggcggcggcggcgg	ggggucggcggcggcggcga	ggggucggcggcggcggcgagccuggcguucgcgggcggcggcggcggcgg
chr1	147994270	147994330	143,4	63 +/- 24%	287	285	1	1	no	gcauaauuugugguaguggggg	uggugcuuuuguggugcg	gcauaauuugugguagugggggacugcguucgcgcuuucccucuggugcuuuuguggugcg
chr15	83424761	83424821	137,1	52 +/- 27%	269	216	0	53	yes	agaccuacuuaucuaccaaca	aagguagauagaacaggucuug	aagguagauagaacaggucuuguuugcaaaauaaauucaagaccuacuuaucuaccaaca
chr15	83424761	83424821	128,9	29 +/- 25%	253	151	0	102	yes	agaccuacuuaucuaccaaca	aagguagauagaacaggucuugu	aagguagauagaacaggucuuguuugcaaaauaaauucaagaccuacuuaucuaccaaca
chr6	41701290	41701348	121,6	58 +/- 8%	235	225	0	10	yes	gcccggagagcugggagccaga	uggcucccuucucuccgucugc	uggcucccuucucuccgucugccuccuggccgcggggcccggagagcugggagccaga
chr15	83424761	83424821	119,2	41 +/- 23%	234	168	0	66	yes	agaccuacuuaucuaccaaca	aagguagauagaacaggucuugu	aagguagauagaacaggucuuguuugcaaaauaaauucaagaccuacuuaucuaccaaca
chr1	147806720	147806778	117,9	77 +/- 6%	229	180	0	49	yes	uccgcugaaaucuugcagugca	gcugcacaauaacaguggaga	gcugcacaauaacaguggagaacugcguuagugcuuuccgcugaaaucuugcagugca
chr9	139964519	139964568	110,8	41 +/- 23%	221	217	0	4	no	gcggcggcggcggcgg	gcggcucgucggccgg	gcggcucgucggccggagcgaacaccaggcgcugcggcggcggcggcgg
chr15	83424762	83424821	110	60 +/- 24%	216	163	0	53	yes	agaccuacuuaucuaccaaca	agguagauagaacaggucuug	agguagauagaacaggucuuguuugcaaaauaaauucaagaccuacuuaucuaccaaca
chr17	19093426	19093483	109,3	52 +/- 38%	221	218	0	3	no	ccacgaggaagagaggua	gccggcuuucuggcguug	ccacgaggaagagagguagcguuuucuccugaacgugaagccggcuuucuggcguug
chr18	8227110	8227176	105,1	59 +/- 16%	205	204	0	1	yes	accacaauuacuuaugcacca	ggugcaaaaguaauugcg	ggugcaaaaguaauugcgacuuuugucguuacuuuuaauggcaaaaccacaauuacuuaugcacca
chr3	46969936	46970001	102	58 +/- 8%	197	183	0	14	yes	aaccacaauuacuuuugcacc	augcaaaaauaacugugguuuu	augcaaaaauaacugugguuuuugccauugaacauaguggcagaaaccacaauuacuuuugcacc
chr15	100047111	100047168	99,4	77 +/- 6%	193	181	0	12	yes	accacaauuacuuuugcacca	gaugcaagaguaauugagguu	gaugcaagaguaauugagguuuucucauuacuuuuaaccacaauuacuuuugcacca
chr17	71640933	71640993	91,9	41 +/- 23%	176	175	0	1	yes	gcgcccggcuccggga	cggagccgagggcgcg	gcgcccggcuccgggacccgcaaaguuugcuagccggcugggcgcggagccgagggcgcg
chr2	233351028	233351096	90,3	59 +/- 16%	181	180	0	1	no	cgcgcgcgccgcucgcuuccu	gccggcggcggcgccu	gccggcggcggcgccugucccgagggcugcccugagcgcaaggccuucgcgcgcgccgcucgcuuccu
chr15	83424761	83424821	81,5	63 +/- 24%	160	138	0	22	yes	agaccuacuuaucuaccaaca	aagguagauagaacaggucuugu	aagguagauagaacaggucuuguuugcaaaauaaauucaagaccuacuuaucuaccaaca
chr13	112276338	112276401	81,4	58 +/- 8%	158	137	0	21	yes	uccacagggucccagauccgagu	acgggguccggauccaagugcacu	acgggguccggauccaagugcacuguuauccucaaauccaguccacagggucccagauccgagu
chr15	83424761	83424821	79,4	59 +/- 31%	156	138	0	18	yes	agaccuacuuaucuaccaaca	aagguagauagaacaggucuug	aagguagauagaacaggucuuguuugcaaaauaaauucaagaccuacuuaucuaccaaca
chr6	41701290	41701348	79,3	59 +/- 16%	152	133	0	19	yes	gcccggagagcugggagccaga	uggcucccuucucuccgucu	uggcucccuucucuccgucugccuccuggccgcggggcccggagagcugggagccaga
chr9	19063656	19063718	77,1	59 +/- 16%	158	123	0	35	no	acugccuuuugaugaccgggacg	uaacggacagauacggggcagaca	acugccuuuugaugaccgggacgaauugagugaaaucguaacggacagauacggggcagaca
chr17	19093425	19093483	73,1	51 +/- 35%	150	149	0	1	no	ccacgaggaagagagg	ggcuuucuggcguugc	ccacgaggaagagagguagcguuuucuccugaacgugaagccggcuuucuggcguugc
chr10	5994723	5994789	70,8	58 +/- 8%	136	122	0	14	yes	ugguaaugaaauaaaaacccugc	aggauuuuuauuucauuacag	aggauuuuuauuucauuacagugugagcugccuggaauacaugugguaaugaaauaaaaacccugc
chr16	85496410	85496471	68,3	17 +/- 26%	137	136	0	1	no	ccgccccccggccccgcgcc	gggaggggccggggcg	gggaggggccggggcgccugggggccccgcagccgcucacgccgccccccggccccgcgcc
chr9	114659094	114659169	66,4	42 +/- 16%	134	61	11	62	no	agugcgcggagcugcggcgguggag	ggcggcggaggugccc	agugcgcggagcugcggcgguggagcugcucggccgggagcccggcggcgagacggagaggcggcggaggugccc
chr4	56262101	56262157	63,7	17 +/- 26%	129	123	0	6	no	gccgccggagaggacggg	gggccgcgccgaggcagcu	gggccgcgccgaggcagcuggcugacuccaguuuagccgccgccggagaggacggg
chr16	30582936	30582998	61,7	59 +/- 16%	116	115	0	1	yes	cgaggccccauggcgccgccccc	cggcggcuccagggaccuggc	cggcggcuccagggaccuggcggccgccgaucggggcugcgaggccccauggcgccgccccc
chr20	26189322	26189369	59,2	59 +/- 16%	112	108	0	4	yes	agcgaggguuccgccggccacc	uggccgagcgcggcucgu	agcgaggguuccgccggccaccgcgguggugggccgagcgcggcucgu
chr11	63568219	63568265	58,6	12 +/- 19%	114	68	0	46	yes	cucacgccuguaauccc	agggcgaggugagcagau	cucacgccuguaaucccaacacuuugggagggcgaggugagcagau
chr2	233215712	233215769	58,2	8 +/- 16%	118	84	0	34	no	cgcgcgcgccgcucgcuucc	cggcgggcggcggcgc	cggcgggcggcggcgccugccccgagugcaaggccuucgcgcgcgccgcucgcuucc
chr11	93463678	93463743	57,4	77 +/- 6%	111	97	1	13	yes	guucucuauaggaagccauag	uguuuuccugaggagauauaaaa	guucucuauaggaagccauagcacuccuaauguuuggugcuauguuuuccugaggagauauaaaa
chr15	83424761	83424821	52,4	12 +/- 33%	103	102	0	1	yes	agaccuacuuaucuaccaaca	aagguagauagaacaggucuugu	aagguagauagaacaggucuuguuugcaaaauaaauucaagaccuacuuaucuaccaaca
chr2	66462425	66462460	51,6	42 +/- 16%	111	110	0	1	no	cacauugccagggauu	ugugugugugugugugu	ugugugugugugugguuucacauugccagggauu
chr1	1063575	1063618	49,7	60 +/- 24%	104	103	0	1	no	gcggcuccgggacggcu	gcgcgcccgccccacc	gcggcuccgggacggcuccccucuccugcgcgcccgccccacc
chr12	123180471	123180533	48,2	58 +/- 8%	92	81	0	11	yes	acccagaaaucugguaugccagc	ugacaagccaggcuucugguc	acccagaaaucugguaugccagcaaagagaguaagaacuucugacaagccaggcuucugguc
chr12	123193896	123193958	48,2	58 +/- 8%	92	81	0	11	yes	acccagaaaucugguaugccagc	ugacaagccaggcuucugguc	acccagaaaucugguaugccagcaaagagaguaagaacuucugacaagccaggcuucugguc

chr12	113729331	113729415	47,8	58 +/- 8%	90	87	0	3	yes	cuggcugcuucccuuggucuccagg	gccggggccauggagc	gccggggccauggagcagccuguguagacggggaccugcccugcaugggcacccccucacuggcugcuucccuuggucuccagg
chr2	88355218	88355276	47,2	12 +/- 19%	89	80	0	9	yes	cggcggaggcggcgguggcg	ucuccgccaccuccaccgcg	cggcggaggcggcgguggcggcagcgguuguuccugccucuccgccaccuccaccgcg
chr5	176566792	176566877	46,5	38 +/- 28%	97	15	75	7	no	cacucaguugguugugguggua	cacuugaggccagaaguu	cacucaguugguugugguggguaugccuguagucccagcuacucaggaggcugaggcaguaguaggaucacuugaggccagaaguu
chr15	83424762	83424821	46,3	48 +/- 23%	91	58	0	33	yes	agaccuacuuaucuaccaaca	agguagauagaacaggucuug	agguagauagaacaggucuuguuugcaaaauaaauucaagaccuacuuaucuaccaaca
chr9	139964518	139964568	45,5	51 +/- 35%	93	91	0	2	no	gcggcggcggcggcgg	cgcggcucgucggccgg	cgcggcucgucggccggagcgaacaccaggcgcugcggcggcggcgg
chr10	124176495	124176565	44,9	58 +/- 8%	85	80	0	5	yes	cacaacugagcaucacagccuga	aggcuaugauccucaguugugu	aggcuaugauccucaguuguguguaaaauuagggcuuucaaucuacacacaacugagcaucacagccuga
chr17	46199545	46199607	43,6	58 +/- 8%	82	74	0	8	yes	agacaguaacagcccgggaca	ccccugggcuguuacuguuccc	agacaguaacagcccgggacagccuuucccaaaauaaaggccccugggcuguuacuguuccc
chr1	166808526	166808583	41,4	58 +/- 8%	85	29	0	56	no	cgggccuggguagccgugcccu	aggcgcggcuuccggccgguc	aggcgcggcuuccggccgguccgggcagucccuggcgggccuggguagccgugcccu
chrY	13258936	13259002	40,7	58 +/- 8%	87	77	1	9	no	acagcaaaggcggagcagug	aucgccagggcggagcugag	aucgccagggcggagcugaguucuccucugcacacacuucggagauacagcaaaggcggagcagug
chr10	99635580	99635641	40,4	58 +/- 8%	76	42	0	34	yes	aggugccauucugagggccagga	ucuggcugcuauggcccccuc	aggugccauucugagggccaggaguuugauuaugugucacucuggcugcuauggcccccuc
chr17	79780678	79780734	38,5	42 +/- 16%	72	51	0	21	yes	acaggacaagggcagccccacc	acugggcugguuuuguccuuga	acugggcugguuuuguccuugaaguggucaggauacaggacaagggcagccccacc
chr2	9590786	9590868	37,7	59 +/- 16%	79	43	20	16	no	gaggcggagguugcagugagccga	gcgcaacugcagucccag	gcgcaacugcagucccagccacucgggaggcugaggcaggagaaucgcuugaaccugggaggcggagguugcagugagccga
chr6	25042370	25042411	35,6	58 +/- 8%	77	58	0	19	no	gcagcugccuucgggccagu	aggcuagggcagcccagca	aggcuagggcagcccagcacagcagcugccuucgggccagu
chr6	32938656	32938714	35,3	77 +/- 6%	65	60	0	5	yes	aggaggggaggagggggagggcu	ccgccgccuccuccgcucgcc	aggaggggaggagggggagggcuagucugagccgcagccgccgccuccuccgcucgcc
chr12	124921988	124922048	34,4	58 +/- 8%	64	46	0	18	yes	cccccuuucccugagccugca	ccaggcucagggaaacugagaca	ccaggcucagggaaacugagacaguggacaagcauugagcccccuuucccugagccugca
chr19	10724867	10724934	34,3	42 +/- 16%	75	54	13	8	no	uccugaccucaggugau	acaggcaugagccaccg	uccugaccucaggugauccaccugccucggccucccaaagugcagggauuacaggcaugagccaccg
chr5	94943814	94943900	34,2	29 +/- 25%	75	73	0	2	no	gucacccaggcuggagug	cuguagcaggguggggag	cuguagcaggguggggaguaaaagggaguaggcuuuuuuuuuuuuuuuuuugagacggagucuugcugugucacccaggcuggagug
chr2	219206639	219206705	33,6	58 +/- 8%	63	47	0	16	yes	uccccugcucccuuguuccccagu	auggggacagggaucagcauggca	auggggacagggaucagcauggcacagauccaauaccuucuguccccugcucccuuguuccccagu
chr12	1914343	1914401	32,4	58 +/- 8%	60	37	0	23	yes	aggacccccccugccuggccuu	gccaggaggaggagguccugu	aggacccccccugccuggccuucgagucuggagggaggccaggaggaggagguccugu
chr7	35841442	35841499	32,2	42 +/- 16%	59	56	0	3	yes	cagucaccgcgucgccuccccg	gguggaggcggcggcggcg	gguggaggcggcggcggcggcugcagccgcagcuccagucaccgcgucgccuccccg
chr22	25416516	25416592	32,1	77 +/- 6%	70	68	0	2	no	gcuaggagcucacagucuagu	gggugaaggggaagca	gcuaggagcucacagucuaguggaggaaacagagguagucaccagguauuccucagugcugggugaaggggaagca
chr2	27274120	27274158	31,8	59 +/- 31%	68	52	0	16	no	gcccgcauccuccacuc	gcgagagguagcggga	gcgagagguagcgggaucgaugcccgcauccuccacuc
chr7	104081757	104081815	31,5	58 +/- 8%	58	33	0	25	yes	cuggcuuguuucauuuagcaua	augcuaaauggaacaagccaagg	augcuaaauggaacaagccaaggacaauuucuguuacuggcuuguuucauuuagcaua
chr14	106843460	106843521	31,4	48 +/- 23%	69	66	0	3	no	ugaaaccccgucucua	ggcgcgguggccagcgccu	ugaaaccccgucucuacuaaaaauacaaaaaaaaauuagcuaggcgcgguggccagcgccu
chr5	176900556	176900614	30,6	41 +/- 23%	65	64	0	1	no	ggcggcggcggcgacu	ccguccgcccguccguccgc	ggcggcggcggcgacucccucuuucccucccuccuccuccguccgcccguccguccgc
chr14	102317618	102317657	30,6	42 +/- 16%	71	50	0	21	no	ccacacccagcuaguu	uagcugggacuacagg	uagcugggacuacaggcaugcacccacacccagcuaguu
chr17	31555852	31555923	30,3	41 +/- 23%	57	56	0	1	yes	ucccuguccuccaggagcu	cuuccaggaggaaaca	cuuccaggaggaaacaaggacacaaagguggcacaccugccucccaaguuccucccuguccuccaggagcu
chr7	129074245	129074339	30,1	41 +/- 23%	55	51	2	2	yes	cccgccgcgccuggga	cggcgcgcggcgguag	cggcgcgcggcgguagggucgccuccggcaaagcgagcugaacccugaggggagccgcugaccagcagcauggaggaccccgccgcgccuggga
chr16	59183155	59183213	29,6	77 +/- 6%	57	55	0	2	yes	gucccaccugggggugc	ccuccaggggacauuu	ccuccaggggacauuugagaacguccggaaauauuuucguugucccaccugggggugc
chr19	19415706	19415765	29,4	77 +/- 6%	55	47	0	8	yes	ccucagaaacgcaccuguuccugu	ggucaggauuguugcugggccca	ccucagaaacgcaccuguuccuguaaacagcaaccaggucaggauuguugcugggccca
chr3	67048783	67048832	29,3	58 +/- 8%	62	44	0	18	no	gccgcgucggcaggugggucgug	guggccagggcggcguggagggag	gccgcgucggcaggugggucgugugguggccagggcggcguggagggag
chr11	268940	269000	29	59 +/- 16%	54	46	0	8	yes	cucacagcuggcgcugaccgcu	uggucagugccuccuguguug	uggucagugccuccuguguuggccgcaaugcugugcuccucacagcuggcgcugaccgcu
chr12	33779828	33779884	29	42 +/- 16%	55	42	0	13	yes	accgcaaccucggucuccugggu	ccaggcuagagugcaaug	ccaggcuagagugcaauggugcgaucuccgcucaccgcaaccucggucuccugggu
chr22	20052703	20052765	28,8	58 +/- 8%	54	37	0	17	yes	ugguccaacgacaggaguaggcu	cccuccucuccuguggccacaga	ugguccaacgacaggaguaggcuuguauuuaaaagcggccccuccucuccuguggccacaga
chr11	64109320	64109382	27,5	77 +/- 6%	49	30	0	19	yes	uggcccaagaccccagaccaca	uggucugaggucuugggccauc	uggucugaggucuugggccaucagugaugucacaaccagauggcccaagaccccagaccaca
chr4	1686018	1686075	27,5	58 +/- 8%	57	32	0	25	no	cgcggcugaggcgcgucgcccugag	ggggcggggcggcggc	ggggcggggcggcggccgcgguuuuguuccgccgcggcugaggcgcgucgcccugag
chr12	26957042	26957106	27,4	77 +/- 6%	53	29	0	24	yes	ucccuuuucugguaguucuca	aggaauuaccugggaaugggaag	aggaauuaccugggaaugggaaguuguuuuguuuuuuagcuuuucccuuuucugguaguucuca
chr1	208042448	208042509	27,3	58 +/- 8%	57	30	0	27	no	cagcggcugcgcaucucgggccugc	acgcccgggaagcggccgccg	acgcccgggaagcggccgccgcggagccggcguccgcagcggcugcgcaucucgggccugc
chr20	37053842	37053913	27	42 +/- 16%	52	35	0	17	yes	aaugaaagcgcuuccccg	ggagcuuucguacaugc	aaugaaagcgcuuccccguguuugaaggguccacuccuaucccuuccaaacucuggagcuuucguacaugc
chr16	33965516	33965580	26,8	63 +/- 24%	58	56	1	1	no	ugccugucugagcgucgcu	ucgacacuucgaacgca	ucgacacuucgaacgcaauugcagcccggguuccucccagggcuuugccugucugagcgucgcu
chr2	219144850	219144907	26,3	58 +/- 8%	57	44	0	13	no	ugggauugacgccacaugucuca	gucaagugucaucugucccua	ugggauugacgccacaugucucaggucccagcugagucaagugucaucugucccua
chr10	9215247	9215305	26,1	59 +/- 16%	49	48	0	1	yes	agccccaauuacuuuugca	aaaaguaauuacaguuuug	aaaaguaauuacaguuuugccauuauugucaaugggaaaagccccaauuacuuuugca
chr6	28106622	28106681	25,9	58 +/- 8%	47	30	0	17	yes	accuagcagaggaaugccaagc	uggcauuccccugcuaggaug	uggcauuccccugcuaggauggaacaguggguuugcaaccuagcagaggaaugccaagc
chr6	121260738	121260797	25,7	77 +/- 6%	47	43	0	4	yes	gauauagauauagauagcccuuga	agggcuaucuauaucuauauc	agggcuaucuauaucuauauccauagauauauauggauauagauauagauagcccuuga
chr6	121260734	121260793	25,7	77 +/- 6%	47	43	0	4	yes	gauauagauauagauagcccuuga	agggcuaucuauaucuauauc	agggcuaucuauaucuauauccauauauaucuauggauauagauauagauagcccuuga
chr8	145317807	145317867	25,4	58 +/- 8%	55	25	0	30	no	cugcgccaggagcucaccaag	uugguggcgucccagcgcagc	uugguggcgucccagcgcagcguccgccucuaccaccugcugcgccaggagcucaccaag
chr8	145486893	145486953	25,4	58 +/- 8%	55	25	0	30	no	cugcgccaggagcucaccaag	uugguggcgucccagcgcagc	uugguggcgucccagcgcagcguccgccucuaccaccugcugcgccaggagcucaccaag
chr11	1498843	1498901	25,3	58 +/- 8%	45	43	0	2	yes	ggcacugaccacgucacuggau	uucagugucuggugagggucccu	ggcacugaccacgucacuggaucugauuagccagauucagugucuggugagggucccu
chr14	93799389	93799448	25,3	42 +/- 16%	53	52	0	1	no	cucgguucggcguggcccu	gccggccggagccccc	cucgguucggcguggcccuggggcgccugcgcggcgcugcggagccggccggagccccc
chr6	43402285	43402360	24,9	58 +/- 8%	46	25	0	21	yes	uggggaaggcuuggcagggaaga	ucccuugucuccuuucccuagc	uggggaaggcuuggcagggaagacacaugagcagugccuccacuucacgccucucccuugucuccuuucccuagc
chr1	234745779	234745857	24,1	58 +/- 8%	51	32	0	19	no	gcggagcgacccggggcccgcgcc	uggccgcugacgcuggcgcugga	uggccgcugacgcuggcgcuggagagcggcgacgccaggagcugugagagagcggcggagcgacccggggcccgcgcc
chr8	52289866	52289906	23,5	42 +/- 16%	52	46	0	6	no	ugagaggugacagcgug	augcuggacugcaguggug	augcuggacugcaguggugcaauugagaggugacagcgug
chr19	19745832	19745882	23,4	58 +/- 8%	51	44	0	7	no	aacaccgugcccuggaugugcagg	ggucacgaagugcacggcu	ggucacgaagugcacggcugagauagaacaccgugcccuggaugugcagg
chr1	52482849	52482886	23,3	42 +/- 16%	44	37	0	7	yes	gccucacugcaaccuc	ugcgugcaguggugcgau	ugcgugcaguggugcgaucucgccucacugcaaccuc
chr6	41701290	41701348	22,4	77 +/- 6%	48	45	0	3	no	gcccggagagcugggagccaga	uggcucccuucucuccgucugccu	uggcucccuucucuccgucugccuccuggccgcggggcccggagagcugggagccaga
chr5	132112882	132112919	22,2	77 +/- 6%	48	41	0	7	no	ccgcccgggcggagcg	cccgcagccggcuggucc	ccgcccgggcggagcgcgucccgcagccggcuggucc
chr2	27274120	27274158	22,1	63 +/- 24%	49	36	0	13	no	gcccgcauccuccacuc	gcgagagguagcggga	gcgagagguagcgggaucgaugcccgcauccuccacuc
chr4	38807015	38807077	22	58 +/- 8%	40	20	0	20	yes	agcguguuuuucacauaggguu	acccuaagugaaaaauaugcu	acccuaagugaaaaauaugcuaagaagaaaagagauucuuagcguguuuuucacauaggguu
chrX	142519397	142519440	21,7	63 +/- 24%	39	21	0	18	yes	ggugcaaaaguauuugugguu	gccgcaauuacuuuugcacc	ggugcaaaaguauuugugguuuugccgcaauuacuuuugcacc
chr2	27274120	27274158	21,6	60 +/- 24%	48	40	0	8	no	gcccgcauccuccacuc	gcgagagguagcggga	gcgagagguagcgggaucgaugcccgcauccuccacuc
chr9	139621267	139621331	21	12 +/- 19%	39	37	0	2	yes	ucugugucauuagguggcagaga	acugccccuagaggcg	acugccccuagaggcguugcagcuguggcugccgugucacaucugugucauuagguggcagaga
chr1	147806720	147806779	21	29 +/- 25%	39	38	0	1	yes	uccgcugaaaucuugcagugca	ggcugcacaauaacaguggaga	ggcugcacaauaacaguggagaacugcguuagugcuuuccgcugaaaucuugcagugca
chr1	174250388	174250451	20,9	29 +/- 25%	48	41	0	7	no	guccauuuuacagagcgcugau	cugcgcccugcugauugguc	cugcgcccugcugauugguccauuuuacagagcacugauugguccauuuuacagagcgcugau
chr2	60622570	60622611	20,9	42 +/- 16%	41	22	0	19	yes	cagcugggugaguggaggca	aacuccauuuacccgu	cagcugggugaguggaggcaugaucaacuccauuuacccgu
chr3	15389121	15389177	20,8	77 +/- 6%	39	31	0	8	yes	ccaacuuugaaguuuuuguca	gacaaaaaccucaaaguua	ccaacuuugaaguuuuugucagauauccaagagaccugacaaaaaccucaaaguua
chr9	42253622	42253686	20,7	77 +/- 6%	47	38	1	8	no	acagcgaagcggagcaug	auugccagggcggagcug	auugccagggcggagcugaguucuccucugcacagacuucggagauacagcgaagcggagcaug
chr9	70648486	70648550	20,7	77 +/- 6%	47	38	1	8	no	acagcgaagcggagcaug	auugccagggcggagcug	auugccagggcggagcugaguucuccucugcacagacuucggagauacagcgaagcggagcaug
chr9	45439867	45439931	20,7	77 +/- 6%	47	38	1	8	no	acagcgaagcggagcaug	auugccagggcggagcug	auugccagggcggagcugaguucuccucugcacagacuucggagauacagcgaagcggagcaug
chr16	845189	845240	20,6	58 +/- 8%	37	36	0	1	yes	uguguggcccucgacuggcugg	gcuggcgggggcugcu	uguguggcccucgacuggcuggccuucgaugaccugcuggcgggggcugcu
chr6	41701289	41701348	20,6	41 +/- 23%	37	35	0	2	yes	gcccggagagcugggagccagag	uggcucccuucucuccgucugcc	uggcucccuucucuccgucugccuccuggccgcggggcccggagagcugggagccagag
chr10	5836868	5836927	20,3	58 +/- 8%	49	46	0	3	no	agugacugaagggagcuuuguc	caagguuccuuccacug	agugacugaagggagcuuugucuauaaggguggaaaaaucuacaagguuccuuccacug
chr7	151217830	151217897	19,8	52 +/- 38%	35	32	0	3	yes	uccgccgcgccgggga	cccggcgugguggcugcu	cccggcgugguggcugcuggagggcuuggcacuugccgaguuagcagagacuccgccgcgccgggga

chr3	49058066	49058129	19,5	77 +/- 6%	35	28	0	7	yes	ggacgaaauccaagcgcagcugg	gcugcuuuugggauuccguugc	ggacgaaauccaagcgcagcuggaaugcucuggagacaacagcugcuuuugggauuccguugc
chr6	105388584	105388631	19,5	30 +/- 23%	42	41	0	1	no	cccuccuccccgcgccc	ggcugcggcggcggagug	cccuccuccccgcgccccgccacucgccgggcugcggcggcggagug
chr9	131314891	131314957	19,5	41 +/- 31%	43	42	0	1	no	cccgacgcacccacgcc	guguggagcggaggccg	guguggagcggaggccgcggaggcuccucgguccuucagcaccccucggcccgacgcacccacgcc
chr14	77495948	77496032	19,3	33 +/- 36%	37	25	8	4	yes	ccagccugggcgacagag	uccgccuucuggggcaaca	ccagccugggcgacagagcaagacuccgucuuaaaaaaaaaaaaaaaaaaaaaaaucacggccuuguuccgccuucuggggcaaca
chr1	25943295	25943399	19	17 +/- 26%	33	26	6	1	yes	gcggcggcggcggcgg	gagcggccgcugcgag	gcggcggcggcggcggggacggcgguggaggcggccggguggcugugcgcgcgugugggcgcaggcucggcggcagcggccggucuggagcggccg
chr15	100047111	100047168	18,8	52 +/- 27%	35	34	0	1	yes	accacaauuacuuuugcacca	gaugcaagaguaauugagguu	gaugcaagaguaauugagguuuucucauuacuuuuaaccacaauuacuuuugcacca
chr12	9680416	9680474	18,4	58 +/- 8%	33	18	0	15	yes	aaugguacuauuuaugcauca	gaugcauaaauaguaccauu	gaugcauaaauaguaccauucucuccuauauauccugaaugguacuauuuaugcauca
chr6	27560499	27560585	18,3	59 +/- 16%	36	20	0	16	yes	agaccuacagugaccgagggagu	uccuccaaaacacugcaucu	uccuccaaaacacugcaucuugcacuuguuagaggugcuuuaacaccucucaugaaugacugaagaccuacagugaccgagggagu
chr1	62152777	62152847	18,2	77 +/- 6%	44	8	35	1	no	caagcuauccugccucggcc	agagacaggaucucacuu	agagacaggaucucacuuuguucccuaggcuggucucaaacuccugggcucaagcuauccugccucggcc
chr12	9680418	9680476	18,2	58 +/- 8%	33	18	0	15	yes	aaugguacuauuuaugcauca	gaugcauaaauaguaccauu	gaugcauaaauaguaccauucaggauauauaggagagaaugguacuauuuaugcauca
chr20	57225268	57225331	18,1	58 +/- 8%	39	34	0	5	no	cccgacccgcuccggccgcgcg	aggcccggggcccggaag	aggcccggggcccggaaggcgccgccgcacagcucugcgcccccgacccgcuccggccgcgcg
chr12	31315701	31315759	18,1	58 +/- 8%	33	18	0	15	yes	aaugguacuauuuaugcauca	gaugcauaaauaguaccauu	gaugcauaaauaguaccauucucuccuauguauccugaaugguacuauuuaugcauca
chr12	57869195	57869257	17,9	58 +/- 8%	31	25	0	6	yes	cgggcgcucacgccccgcucua	cgggcuggggagggagag	cgggcuggggagggagaguccgggagcgcagcgaggcucccgggcgcucacgccccgcucua
chr3	187446942	187447008	17,8	8 +/- 16%	40	38	0	2	no	ugagcaggcugagcugggccgccuu	acacggccccaccugccugccagccacc	ugagcaggcugagcugggccgccuuuccccacgagccuacacggccccaccugccugccagccacc
chr12	31315699	31315757	17,8	58 +/- 8%	33	18	0	15	yes	aaugguacuauuuaugcauca	gaugcauaaauaguaccauu	gaugcauaaauaguaccauucaggauacauaggagagaaugguacuauuuaugcauca
chr3	122692547	122692608	17,7	77 +/- 6%	31	25	0	6	yes	ugcccuccuugggaaucuacaa	gcagauucccagagugggacagg	gcagauucccagagugggacagggaggcuccacauugccugcccuccuugggaaucuacaa
chr14	77495016	77495091	17,6	41 +/- 31%	38	36	0	2	no	gcccgggcggagcgcuga	ggcgcuccugcuccgg	ggcgcuccugcuccggcugcggcucgcgcuguccccggcuuggccgcgcgggaugccgcccgggcggagcgcuga
chr19	12753429	12753495	17,5	58 +/- 8%	39	32	0	7	no	uccggccggagagcccaggacag	uggcaaggucuucucggccgg	uccggccggagagcccaggacaguguuccaggagaccgaaggagauggcaaggucuucucggccgg
chr1	153508755	153508810	17,5	58 +/- 8%	39	33	0	6	no	ucaccccugcuuccucugccaga	cggcaaggggagcaggagga	cggcaaggggagcaggaggaggacugcggcggucaccccugcuuccucugccaga
chr7	99954278	99954341	17,3	77 +/- 6%	31	22	0	9	yes	cacccccgggcaaagaccugcagau	gcccaggacuuugugcggggug	cacccccgggcaaagaccugcagauccccuguuagagacgggcccaggacuuugugcggggug
chr13	35537588	35537648	17,2	77 +/- 6%	30	27	0	3	yes	uccccccaaaauucauaauguu	gcauaugaauuuuuggggggau	uccccccaaaauucauaauguugaaguccucaccuuuagcauaugaauuuuuggggggau
chr4	129732848	129732887	16,7	77 +/- 6%	36	7	0	29	no	cccgcggcgcgggggguggccg	ugcgcccccgccccggg	cccgcggcgcgggggguggccgcgcugcgcccccgccccggg
chr6	41701289	41701350	16,6	60 +/- 24%	29	24	0	5	yes	gcccggagagcugggagccagag	ccuggcucccuucucuccguc	ccuggcucccuucucuccgucugccuccuggccgcggggcccggagagcugggagccagag
chr2	43451498	43451549	16,5	8 +/- 16%	37	33	0	4	no	gccgcugaggcugccggag	cggcggccagggucga	cggcggccagggucgaggcugggagacucagagccgcugaggcugccggag
chr5	96459136	96459189	16,5	59 +/- 16%	29	20	0	9	yes	gagacuucauuaggcaugaaagu	uuucauuccuagugaaguc	gagacuucauuaggcaugaaaguaucuucccaacuuucauuccuagugaaguc
chr19	13263869	13263908	16,5	42 +/- 16%	37	24	0	13	no	agagcguccuagcagug	cugcgugggguugguuugug	agagcguccuagcagugucacugcgugggguugguuugug
chr1	166808741	166808825	16,2	59 +/- 31%	36	17	0	19	no	ccgcgcgcacgggagg	ccccaccccgcgcccu	ccgcgcgcacgggaggacggagaggcggaaaggauggcgcugugacagccgggccggagcccucgcgucccccaccccgcgcccu
chr17	7210152	7210207	15,9	77 +/- 6%	28	24	0	4	yes	acccucaguccguauuggucucu	gaggccauucggcucugagg	acccucaguccguauuggucucuauggcauccauagaggccauucggcucugagg
chr3	155027516	155027569	15,7	77 +/- 6%	35	15	0	20	no	accccgacgugaccgaggcgaugcu	auggccucgcucuacgugggggac	auggccucgcucuacgugggggaccuccaccccgacgugaccgaggcgaugcu
chr7	153321297	153321339	15,6	42 +/- 16%	38	36	0	2	no	agggacagagagguugag	ggcacgucuucugcuu	ggcacgucuucugcuucuucaacaagggacagagagguugag
chr3	32547793	32547859	15,5	52 +/- 27%	27	10	0	17	yes	aaccgcgauuacucuugcacga	ggugcaaaaguaauugugguu	ggugcaaaaguaauugugguuuuugcauuuaaaguaauggccaaaaccgcgauuacucuugcacga
chr14	96000143	96000203	15,4	77 +/- 6%	28	25	0	3	yes	ugcagaggagacaaagcaag	uugcucugcucucccuuguacu	ugcagaggagacaaagcaagccucaucaggcccauugcuugcucugcucucccuuguacu
chr10	31892411	31892494	15,4	58 +/- 8%	34	31	2	1	no	gggaaggggcguggcccugc	uggccgcccucccuccucc	gggaaggggcguggcccugcccgagcuggcgccagggaggcucccaccugccagggguuguacauggccgcccucccuccucc
chr8	101733731	101733784	15,4	77 +/- 6%	35	15	0	20	no	accccgacgugaccgaggcgaugcu	auggccucgcucuacgugggggac	auggccucgcucuacgugggggaccuccaccccgacgugaccgaggcgaugcu
chr13	86047061	86047102	15,3	59 +/- 16%	28	14	0	14	yes	uggcgccacugcacuccagc	ggagcuugcagugagccga	ggagcuugcagugagccgagauggcgccacugcacuccagc
chr9	117379978	117380073	15,3	38 +/- 28%	29	16	8	5	yes	guugcccaggcuggucuca	acagcugggauuacaggca	acagcugggauuacaggcauccaccgcuacauccugcuaauuaaaaauguuuuuuguagaguuggggccuuacuuuguugcccaggcuggucuca
chr12	121442296	121442362	15,1	58 +/- 8%	34	18	0	16	no	gacagcgagaggcagcucugggg	caggguacccucucguugca	gacagcgagaggcagcucuggggcuguguccugacuccagcuaacccaggguacccucucguugca
chr2	85109655	85109714	14,8	77 +/- 6%	26	12	0	14	yes	ugggcuucucucuggccacaca	guggccacagagaagcc	guggccacagagaagcccgcccagcuucucucugagcugggcuucucucuggccacaca
chr3	88108072	88108116	14,7	30 +/- 23%	33	24	0	9	no	cggcggcgacggcggc	gcggccgccgcggggc	gcggccgccgcggggcucgaucgggcaacggcggcgacggcggc
chr3	41274854	41274911	14,4	58 +/- 8%	38	24	0	14	no	ucaccugacagauccaagucaacg	acugucuuuggacucucaggaa	ucaccugacagauccaagucaacgucuuguucagaacugucuuuggacucucaggaa
chr2	22518111	22518168	14,4	33 +/- 36%	26	25	0	1	yes	cacccaggcuggagugcagugg	cacugcaaccuccaccu	cacccaggcuggagugcaguggagcgaucucgacucacaucacugcaaccuccaccu
chr9	19063657	19063718	14,2	77 +/- 6%	27	24	0	3	yes	acugccuuuugaugaccgggacg	uaacggacagauacggggcagac	acugccuuuugaugaccgggacgaauugagugaaaucguaacggacagauacggggcagac
chr9	19063656	19063718	14,2	41 +/- 31%	27	18	0	9	yes	acugccuuuugaugaccgggacg	uaacggacagauacggggcagaca	acugccuuuugaugaccgggacgaauugagugaaaucguaacggacagauacggggcagaca
chr7	44028715	44028772	14	77 +/- 6%	26	25	0	1	yes	uggaguugaguugaauucaacg	ugaacugaacugaacugaaca	uggaguugaguugaauucaacguaaaugaaucaaguugaacugaacugaacugaaca
chr7	102182978	102183035	14	77 +/- 6%	26	25	0	1	yes	uggaguugaguugaauucaacg	ugaacugaacugaacugaaca	uggaguugaguugaauucaacguaaaugaaucaaguugaacugaacugaacugaaca
chr7	102282089	102282146	14	77 +/- 6%	26	25	0	1	yes	uggaguugaguugaauucaacg	ugaacugaacugaacugaaca	uggaguugaguugaauucaacguaaaugaaugaaguugaacugaacugaacugaaca
chr3	23852240	23852305	14	60 +/- 24%	31	29	0	2	no	ccccccaccccgcgcgcg	gcgggcgggggagggg	cccccaccccgcgcgcgcccccgacggcucgcgccgcgccggugaaguugcgggcgggggagggg
chr10	124176495	124176565	13,8	59 +/- 16%	24	19	0	5	yes	cacaacugagcaucacagccuga	aggcuaugauccucaguugugu	aggcuaugauccucaguuguguguaaaauuagggcuuucaaucuacacacaacugagcaucacagccuga
chr1	160040795	160040856	13,7	58 +/- 8%	24	20	0	4	yes	gguccccaggacucuguugcau	cguagcagaguccuggggaga	gguccccaggacucuguugcauuuaaggcagagggaacaacguagcagaguccuggggaga
chr12	110906561	110906615	13,7	77 +/- 6%	23	22	0	1	yes	cccgcucucccgaagcuguuc	aacggcuucggaggggcgaga	cccgcucucccgaagcuguucgggcaguguccgaacggcuucggaggggcgaga
chr11	57296398	57296468	13,6	59 +/- 16%	32	27	0	5	no	agcagggcuggaauggucaagguu	accuugccccucccuc	agcagggcuggaauggucaagguuccugggaugucacaggaugcugcugacaugaccuugccccucccuc
chr16	81041261	81041314	13,6	58 +/- 8%	33	19	0	14	no	acccucuugcguugcuccu	gagcgcagcagaggugugug	acccucuugcguugcuccucgcauugccuguuggagcgcagcagaggugugug
chr1	109289267	109289349	13,5	30 +/- 23%	23	12	7	4	yes	gugguggcugcugcuccgca	gcagcaaggggcggggc	gcagcaaggggcggggccaccccaacgccgcuucugcggccaaaguagguugggaguggaagguggugggcugcugcuccgca
chr1	166808526	166808583	13,4	77 +/- 6%	30	25	0	5	no	aggcgcggcuuccggccgguc	cgggccuggguagccgugcccu	aggcgcggcuuccggccgguccgggcagucccuggcgggccuggguagccgugcccu
chr17	73106348	73106422	13,2	77 +/- 6%	22	21	0	1	yes	cccgcccgucccugggggguccuc	ggacccccacgucgggcg	cccgcccgucccugggggguccuccgucaccgcggccauggcccagaagccgaagguggacccccacgucgggcg
chr6	41701290	41701348	13	52 +/- 27%	22	11	0	11	yes	uggcucccuucucuccgucug	gcccggagagcugggagccaga	uggcucccuucucuccgucugccuccuggccgcggggcccggagagcugggagccaga
chr17	48943678	48943731	12,9	60 +/- 24%	30	27	0	3	no	gggcgcgcgccgcggcag	cccggcguguagcgggcg	gggcgcgcgccgcggcagaaggagcgaagcucuggcccggcguguagcgggcg
chr1	227941452	227941506	12,9	77 +/- 6%	21	16	0	5	yes	cuggcuucagugcccuguuga	gcagggcacugaagccagcc	gcagggcacugaagccagccguggcuucaguggcuggcuucagugcccuguuga
chr18	53146457	53146518	12,8	77 +/- 6%	20	17	0	3	yes	ggccaucagcaguccaaugaaga	cuucauuggacugcugauggcc	ggccaucagcaguccaaugaagacauugggucuucaugucuucauuggacugcugauggcc
chr19	14800870	14800928	12,8	12 +/- 19%	30	29	0	1	no	cggccgccgccgcgcg	ugccgcaccgcgccgcgcc	ugccgcaccgcgccgcgccgcgcagcacagcucagccgccgucggccgccgccgcgcg
chr17	41154689	41154756	12,7	38 +/- 28%	23	9	0	14	yes	gguacucuguggauaucccccu	aggaugucuucagagauccugcucu	gguacucuguggauauccccuuggacaaaacugucgucaauaaggaugucuucagagauccugcucu
chr15	83424761	83424821	12,7	23 +/- 42%	25	22	0	3	yes	agaccuacuuaucuaccaaca	aagguagauagaacaggucuug	aagguagauagaacaggucuuguuugcaaaauaaauucaagaccuacuuaucuaccaaca
chr22	24407606	24407696	12,6	48 +/- 23%	28	23	2	3	no	gcacgcgccgcucgcu	gcgugcgcgcgugugu	gcacgcgccgcucgcucuccgcgggcugcuuagcuggggcccugcagacggcuacccuugggcggcgcgcgcgcgcgugcgcgcgugugu
chr7	44112975	44113027	12,6	77 +/- 6%	22	18	0	4	yes	aaguccugcuucuguugcaga	aagcagcaguggcaagacu	aagcagcaguggcaagacuccuaggucacggaaguccugcuucuguugcaga
chr2	155332361	155332433	12,5	30 +/- 23%	37	36	0	1	no	auuccacuucugacacca	gggccgagugggaacca	gggccgagugggaaccacgcucagcgcuccugggggguuguuagcucuucuuuggauuccacuucugacacca
chr7	44364884	44364948	12,4	52 +/- 27%	28	27	0	1	no	ccagcccgcgcgcgccgcu	cggcgcggagggucccgg	cggcgcggagggucccggaggucccggcccucuggggucccggcccagcccgcgcgcgccgcu
chr2	80914301	80914343	12,3	42 +/- 16%	24	22	0	2	yes	ugcuugagcucaggaguu	cccgccugagcaacauagc	ugcuugagcucaggaguucaagacccgccugagcaacauagc
chr14	55311011	55311068	12,3	77 +/- 6%	23	12	0	11	yes	aaagauaauugcaguuuuugcc	caagaaccgcaguuacuuuug	aaagauaauugcaguuuuugccauuauguucaauagcaagaaccgcaguuacuuuug
chr15	69109035	69109088	12,1	77 +/- 6%	21	19	0	2	yes	gcuggugagcuuaaccuaccu	uaggauacguuuaccugcccuu	uaggauacguuuaccugcccuugugaccuugggcuggugagcuuaaccuaccu
chr17	33760234	33760292	12	77 +/- 6%	21	16	0	5	yes	uccacacuuccccagcguggccug	gcuggcgcuccggaagugu	uccacacuuccccagcguggccugcauuucuauugggcagcuggcgcuccggaagugu
chr11	93463678	93463743	12	29 +/- 25%	22	21	0	1	yes	guucucuauaggaagccauag	uguuuuccugaggagauauaaaa	guucucuauaggaagccauagcacuccuaauguuuggugcuauguuuuccugaggagauauaaaa
chr4	88946643	88946708	12	48 +/- 23%	21	9	6	6	yes	guagucccagcuacucagg	uggaagauggagacugca	guagucccagcuacucaggaggcugaggcaagaggaugacuugagucuggaagauggagacugca

chr8	12883906	12883978	11,9	59 +/- 16%	20	15	0	5	yes	gggguggggggggaggg	cucuugcccucuacccccg	cucuugcccucuacccccggggcuuguauauagauuauaaauauauaaggggggaaaggggugggggggaggg
chr20	30449496	30449555	11,9	17 +/- 26%	19	13	0	6	yes	cgccggccuugaccccgcccac	gggugggggcggggccggcugcc	gggugggggcggggccggcugccuugaccccgccccgcgccggccuugaccccgcccac
chr20	48453822	48453880	11,9	30 +/- 23%	21	12	0	9	yes	cgaccaccuugggcacaugucg	ggcugugucaaggguggucauc	cgaccaccuugggcacaugucgucaggaccuccugaggcugugucaaggguggucauc
chr3	67096312	67096371	11,9	77 +/- 6%	20	17	0	3	yes	augcaaaucugcaaaauaaccu	auuauuuugcagauuugcaugu	augcaaaucugcaaaauaaccuugguauuuugccaagauuauuuugcagauuugcaugu
chr17	41154690	41154753	11,6	58 +/- 8%	21	15	0	6	yes	guacucuguggauaucccc	gaugucuucagagauccugc	guacucuguggauauccccuuggacaaaacugucgucaauaaggaugucuucagagauccugc
chr3	127305953	127306019	11,5	38 +/- 28%	19	16	0	3	yes	agaucaaaagccucagggcaga	ugcccugagacuuuugcucuaa	ugcccugagacuuuugcucuaauaauuuauucuaauaauaauuuagaucaaaagccucagggcaga
chr2	120750871	120750934	11,3	58 +/- 8%	21	15	0	6	yes	guacucuguggauaucccc	gaugucuucagagauccugc	guacucuguggauauccccuuggacaaaacuguugucaguaacgaugucuucagagauccugc
chr16	18877124	18877187	11,1	42 +/- 16%	34	25	0	9	no	ugugguggcacauacgug	ggugaaacccugucucua	ggugaaacccugucucuacuaaaaauacaaaaaauauuagcgaggugugguggcacauacgug
chr7	99036583	99036642	11	17 +/- 26%	27	17	9	1	no	ggcggcggcggcggcg	ggcggggccggcggcgg	ggcggcggcggcggcgaggcgaagcgaaggaggagugugugcggcggggccggcggcgg
chr3	107553216	107553275	10,9	77 +/- 6%	19	18	0	1	yes	gaagaugcccagggguucuga	gcgaucccuugagcauuc	gcgaucccuugagcauucuccuuaagguggugcuuagaagaagaugcccagggguucuga
chrX	49113328	49113381	10,9	77 +/- 6%	19	12	0	7	yes	cuccuaucccucccccugaca	gucaguggacagggcugggaaggauc	gucaguggacagggcugggaaggauccucgcccuccuaucccuccccugaca
chr4	6829968	6830046	10,7	38 +/- 28%	27	10	14	3	no	ggguuuaccuguagucccagcaac	gguggaggcugcagug	ggguuuaccuguagucccagcaacuugggaagcugagguaggaggaucgcuugagccugggggguggaggcugcagug
chr20	51728376	51728433	10,7	58 +/- 8%	17	16	0	1	yes	agcuuacaauccuuggggguc	acccccagagauugca	acccccagagauugcacgcucugguaccugaugcagagcuuacaauccuuggggguc
chr8	11058666	11058738	10,5	30 +/- 23%	17	10	0	7	yes	gccgccgccgccgcagcc	guugcagcaguggcagau	guugcagcaguggcagaugugcaucgagcugcucucgccgggcucgcugccgucgccgccgccgccgcagcc
chr11	8932707	8932783	10,4	58 +/- 8%	25	24	0	1	no	gugagccgggugagggggcuc	gcgcuaggccuggucc	gcgcuaggccuggucccucuuccuagaauagcguugcgcgcaugcgccuugacgagugagccgggugagggggcuc
chr19	30433171	30433208	10,4	30 +/- 23%	24	19	0	5	no	gacgccuggcugggccc	accggagaggcgucucgg	gacgccuggcugggcccgcaccggagaggcgucucgg
chr6	117996996	117997069	10,3	58 +/- 8%	24	18	0	6	no	gccaggcacgaccccccgcg	gccgggggcuugcggagcg	gccaggcacgaccccccgcgcgggugccgguggugacggcgguuccugccgacugccgggggcuugcggagcg
chrX	47372647	47372720	10,3	58 +/- 8%	24	18	0	6	no	gccaggcacgaccccccgcg	gccgggggcuugcggagcg	gccaggcacgaccccccgcgcgggugccgguggugacggcgguuccugccgacugccgggggcuugcggagcg
chr19	45492635	45492693	10,3	77 +/- 6%	17	10	0	7	yes	gcaggccucacuuggcaccagug	ccuggucuaaggagaggcuuggg	gcaggccucacuuggcaccagugcuguguggccucccuggucuaaggagaggcuuggg
chr15	69978094	69978153	10,1	77 +/- 6%	17	6	0	11	yes	cccugagggugagugccucc	gaggcacugacucucagggcu	gaggcacugacucucagggcucagccuguauucccacagcccugagggugagugccucc
chr16	47177853	47177957	10	30 +/- 23%	23	14	8	1	no	gcgcggggcggggcgg	ccccgcgcccgccacu	gcgcggggcggggcggggcgggcacaugcgcacaggcggcgcagccggcacgcggcgcucgcgcucccuccuuaaaugagccugggcgccccgcgccc
chr5	133930901	133930955	10	77 +/- 6%	16	13	0	3	yes	agucagccacucagcaggcacu	cugccugcugguggcuggcaug	agucagccacucagcaggcacuuccugaacaccugccugcugguggcuggcaug
chr17	40688487	40688556	9,9	37 +/- 22%	23	16	0	7	no	gccugggcggcggcggcg	gcggccgccgcggggc	gccugggcggcggcggcgcggcgcgcgugcgggugcgcggcuccacgggcguggcggccgccgcggggc
chr10	124176495	124176567	9,6	43 +/- 22%	16	10	0	6	yes	ucaggcuaugauccucaguugug	acaacugagcaucacagccuga	ucaggcuaugauccucaguuguguaaaauuagggcuuucaaucuacacacaacugagcaucacagccuga
chr17	7809562	7809638	9,6	37 +/- 25%	25	21	1	3	no	ggaaaggaugaagagcugacugaug	guccuagccucacauu	guccuagccucacauuuacuugaccacaauaaccuggcaggaggagcugguggaaaggaugaagagcugacugaug
chr7	77325778	77325816	9,5	37 +/- 25%	24	17	0	7	no	gccgagccccgugcacug	gagcgcaaaauggcggaa	gagcgcaaaauggcggaaccgccgagccccgugcacug
chr11	67084969	67085025	9,5	77 +/- 6%	15	14	0	1	yes	ccucgcccuagccagggcugc	agcccaggcuggggcggggccu	agcccaggcuggggcggggccucggaagcaacugcccucgcccuagccagggcugc
chr6	410518	410595	9,4	77 +/- 6%	16	13	0	3	yes	uaccaccaagggcagguagguauu	auacagcuagccccag	uaccaccaagggcagguagguauuaguguuugaaaaagcuggucuuugagcgagggcauaaauacagcuagccccag
chrX	153200619	153200679	9,4	19 +/- 22%	22	21	0	1	no	ggccugggggcggggc	ccccucccucggcgcgac	ggccugggggcggggcgggcgcugccccggagcuggagccggccccucccucggcgcgac
chr6	41701289	41701348	9,4	50 +/- 22%	15	13	0	2	yes	gcccggagagcugggagccagag	uggcucccuucucuccgucugcc	uggcucccuucucuccgucugccuccuggccgcggggcccggagagcugggagccagag
chr2	24583319	24583390	9,3	37 +/- 22%	22	17	0	5	no	ggcuacgcaacagguucg	cauguuguuguggggcuga	cauguuguuguggggcugaggcggcgccggcggagcccugagcggcugugacaggcuacgcaacagguucg
chr4	86594817	86594872	9,2	43 +/- 15%	13	12	0	1	yes	ccagugggggggaucuaagcauu	augcuuagaucuccccacuggua	augcuuagaucuccccacugguaaagugaaacaccaguggggggaucuaagcauu
chr7	103848477	103848558	9,1	56 +/- 8%	22	18	0	4	no	cuagucucagggccggaacca	gagccugcgcugccgggcu	gagccugcgcugccgggcugcggcgugacggcagcgcguacgccgagcgucgccgccgcucuagucucagggccggaacca
chr7	44112975	44113027	9,1	37 +/- 22%	15	10	0	5	yes	aaguccugcuucuguugcaga	aagcagcaguggcaagacu	aagcagcaguggcaagacuccuaggucacggaaguccugcuucuguugcaga
chr2	96656064	96656125	9	77 +/- 6%	14	9	0	5	yes	cgaaaacugcaauugccuuugc	aaaagcaauugcgguuuuug	aaaagcaauugcgguuuuugccgcaauuacaaguaauggcgaaaacugcaauugccuuugc
chr19	45844819	45844854	8,9	67 +/- 21%	15	14	0	1	yes	cgcgcgcgcguguggu	gugugugugugugugug	gugugugugugugugugcgcgcgcgcguguggu
chr12	12224339	12224425	8,9	57 +/- 8%	28	24	0	4	no	aaugacaugacagccauuccgug	auuacugguugaggua	auuacugguugagguaugugacguuacucagucuucucugcuuauguaucuggggagguaugcaaugacaugacagccauuccgug
chr15	85176756	85176827	8,7	57 +/- 8%	21	20	0	1	no	cggcagccgagggggccggu	cugcgcuccuccgcagccug	cugcgcuccuccgcagccugggccgggccgcccgggacgcugaggcgguggcggcagccgagggggccggu
chr4	109542558	109542628	8,7	46 +/- 15%	24	18	0	6	no	aggcauuguguugggacg	gccuuuacgcagguggug	gccuuuacgcagguggugcuuuagggcaagacauugaacccugauaugugccaggcauuguguugggacg
chr2	121832864	121832932	8,6	57 +/- 8%	24	20	1	3	no	uugcugcguggacugugcu	auagcugcacgcggcgu	uugcugcguggacugugcuggggggggucagggggcagauaaaugaaagaggauagcugcacgcggcgu
chr17	62007748	62007809	8,5	15 +/- 23%	21	16	0	5	no	cugcuggggcuggcacucagga	cugagccuggccuuggcaccca	cugcuggggcuggcacucaggaagaugccaagcggaaugcugagccuggccuuggcaccca
chr20	35926506	35926575	8,4	62 +/- 29%	14	13	0	1	yes	ccagccugggugacagag	uugagcauaagaguuugaggcua	uugagcauaagaguuugaggcuacagugaguuauguuugugccauuguacuccagccugggugacagag
chr17	38210583	38210635	8,3	32 +/- 21%	16	13	0	3	yes	ccaccuagaacuggauugugc	aaauuugggaggugguggaug	ccaccuagaacuggauugugcgguaggcuuaaauuugggaggugguggaug
chr19	52725327	52725378	8,2	75 +/- 6%	22	20	0	2	no	accccugccacucacuggccccc	gaggucugugggcagga	accccugccacucacuggcccccaggugcugucugaggucugugggcagga
chr16	33966073	33966116	8,1	46 +/- 15%	12	11	0	1	yes	gccgcgguucgccgcc	cgguccggcgaccggc	gccgcgguucgccgccgccccugguggcgguccggcgaccggc
chr21	9826615	9826703	8,1	46 +/- 29%	19	13	3	3	no	ccgggcgcgggucggg	guccccgccucgccgc	guccccgccucgccgccgcccgcgggcgccggccgcgcgcgcgcgcguggccgccggucccucccggccgccgggcgcgggucggg
chr15	63610837	63610894	8	75 +/- 6%	11	10	0	1	yes	ccucccacccacagggcgagucc	acucgcccuaugggugggagc	ccucccacccacagggcgaguccaagggaaguauggacucgcccuaugggugggagc
chr7	23513876	23513954	8	38 +/- 25%	20	15	0	5	no	ccccggggagcgcgggagc	cccgcagacccgcaggg	ccccggggagcgcgggagccccgccuuggagccgcuuuugcgcgccagucucgcggcccgccccgcagacccgcaggg
chr2	64567901	64567963	8	35 +/- 26%	11	10	0	1	yes	acaggaguggggggugggacgua	augucccacccccacuccuguu	augucccacccccacuccuguuugaauauuucaccagaaacaggaguggggggugggacgua
chr6	283317	283378	7,9	75 +/- 6%	14	12	0	2	yes	ugaagaucaucagaugcuagu	acgcauucagugaucuuucgc	ugaagaucaucagaugcuagucuaagccacuuagcaacggacgcauucagugaucuuucgc
chr7	44079261	44079341	7,9	63 +/- 12%	20	19	0	1	no	uccuuccugcugucaccuccagg	ggggaggggggguggg	ggggagggggggugggagggggugcaguggcuaggccccuaaccuagcagaaccugguccuuccugcugucaccuccagg
chr7	102134415	102134495	7,9	63 +/- 12%	20	19	0	1	no	uccuuccugcugucaccuccagg	ggggaggggggguggg	ggggagggggggugggagggggugcaguggcuaggccccuaaccuagcagaaccugguccuuccugcugucaccuccagg
chr7	102233584	102233664	7,9	63 +/- 12%	20	19	0	1	no	uccuuccugcugucaccuccagg	ggggaggggggguggg	ggggagggggggugggagggggugcaguggcuaggccccuaaccuagcagaaccugguccuuccugcugucaccuccagg
chr7	102329494	102329574	7,9	63 +/- 12%	20	19	0	1	no	uccuuccugcugucaccuccagg	ggggaggggggguggg	ggggagggggggugggagggggugcaguggcuaggccccuaaccuagcagaaccugguccuuccugcugucaccuccagg
chr12	111021030	111021105	7,9	62 +/- 22%	19	18	0	1	no	ggcggcggcggcggcgg	gccgccgcuucugaccucg	ggcggcggcggcggcggccgcugcagccgggacuucaguugggcugaggcggaggagccgccgcuucugaccucg
chr17	46199547	46199606	7,9	32 +/- 24%	12	11	0	1	yes	gacaguaacagcccgggacagc	ccccugggcuguuacuguuc	gacaguaacagcccgggacagccuuucccaaaauaaaggccccugggcuguuacuguuc
chr19	7533878	7533930	7,9	27 +/- 34%	21	20	0	1	no	ccagcugcucagcgccacca	ggcggccgugcagcag	ccagcugcucagcgccaccaaccaguuccagaggcaggcggccgugcagcag
chr20	30449497	30449555	7,8	52 +/- 31%	11	7	0	4	yes	cgccggccuugaccccgccca	gggugggggcggggccggcugcc	gggugggggcggggccggcugccuugaccccgccccgcgccggccuugaccccgccca
chr5	141016344	141016420	7,7	63 +/- 12%	19	11	4	4	no	gcaccauggccaagaccg	aggggcuggcggcggc	aggggcuggcggcggccgcgggcggcgggcggcggaggugcggggccugcucccgccggcaccauggccaagaccg
chr13	45986480	45986538	7,7	35 +/- 20%	21	13	0	8	no	cagcacuuugggaggcu	agccugugguggcugggcau	agccugugguggcugggcauaguggcucucaccucuuuucccagcacuuugggaggcu
chr7	100027681	100027731	7,6	35 +/- 20%	11	10	0	1	yes	gucgggcggagggggcggccc	ccggccccgccgccgc	ccggccccgccgccgccgcucaaagaugagucgggcggagggggcggccc
chr1	12040457	12040545	7,6	19 +/- 23%	19	13	4	2	no	cgaggccgggaaggugaaggug	cuucccuccucccgccu	cuucccuccucccgccuccccccuggggguggcgcucgcuggugacguagugagugugauggccgccgcgaggccgggaaggugaaggug
chr12	58149381	58149430	7,6	63 +/- 12%	10	9	0	1	yes	ccccgggccgagccgcaaccccg	ggaggcggcugcggcu	ccccgggccgagccgcaaccccggggggggccggggaggcggcugcggcu
chr9	37374527	37374580	7,5	56 +/- 7%	23	22	0	1	no	guuggauggggacacagaca	gccugcacccccagacc	gccugcacccccagaccaauggcaucggaaucuguuggauggggacacagaca
chrX	152600928	152600989	7,5	75 +/- 6%	11	9	0	2	yes	ggauguacugaacugggucaga	uggcccaguucaguacauc	uggcccaguucaguacauccccacaaaauguuacuaggggauguacugaacugggucaga
chr11	16759717	16759775	7,4	46 +/- 15%	25	24	0	1	no	accauuuccacccuggug	cuagguggucggugau	accauuuccacccugguguucucuauugcgcgcaggggauuucuagguggucggugau
chr6	11255896	11255952	7,2	75 +/- 6%	10	6	0	4	yes	gauccaugcaaggccuuuugggg	caaaaggccucgcauggcucca	caaaaggccucgcauggcuccagcggcuauguggauccaugcaaggccuuuugggg
chr4	86594814	86594870	7,2	46 +/- 26%	9	8	0	1	yes	caguggggagaucuaagcauug	augcuuagaucccccacuggu	augcuuagaucccccacugguguuucacuuuaccaguggggagaucuaagcauug
chr15	101583484	101583520	7,1	75 +/- 6%	10	7	0	3	yes	gggagguggguggggccu	gccccucccauaccua	gccccucccauaccuagugggagguggguggggccu
chr1	225615997	225616064	7,1	35 +/- 20%	19	12	0	7	no	cucgcagcacacggagcc	gcccggagcgcgacguuugcc	gcccggagcgcgacguuugccaccgccuuaucucagccgcccaggcgcccucgcagcacacggagcc
chr19	5978316	5978373	7,1	75 +/- 6%	10	7	0	3	yes	cggggaaagggccgggaagggc	caucucccgccuccuuuucccgcc	caucucccgccuccuuuucccgccgacugagagggcggggaaagggccgggaagggc
chr11	82867686	82867730	7,1	19 +/- 23%	10	6	0	4	yes	ccgcacccccucuccc	gguuggagggagcggaggcu	gguuggagggagcggaggcuuucugccgccgcacccccucuccc

chr10	124176495	124176566	7,1	62 +/- 22%	11	9	0	2	yes	cacaacugagcaucacagccuga	caggcuaugauccucaguugug	caggcuaugauccucaguuguguguaaaauuagggcuuucaaucuacacacaacugagcaucacagccuga
chr16	2587093	2587138	7,1	24 +/- 24%	21	15	0	6	no	aucacuggaguuggagac	gccaacauggugaaaccc	aucacuggaguuggagaccagccccuggccaacauggugaaaccc
chr1	154572323	154572384	7	75 +/- 6%	11	9	0	2	yes	ugccugccuguugaagguggugg	cuccucugcacagaugggguagg	ugccugccuguugaagguggugguucuaauuuuauaaaccuccucugcacagaugggguagg
chrX	118533316	118533363	7	62 +/- 22%	9	6	0	3	yes	gagagccccaggcccgagccacgc	cgggcccgggucgggg	gagagccccaggcccgagccacgcggucuuccgggcccgggucgggg
chr17	63052672	63052732	6,9	61 +/- 27%	17	13	0	4	no	ggcggcggcggcggcg	cgcgguccgugcuguccgug	ggcggcggcggcggcggcaagauggcggacuuccugccgucgcgguccgugcuguccgug
chr19	36540022	36540071	6,9	40 +/- 34%	19	17	0	2	no	cccagcuacucaggaggc	ugguccgaguguugugggguu	cccagcuacucaggaggcugagauggagcugguccgaguguugugggguu
chr15	101583484	101583520	6,8	74 +/- 6%	10	7	0	3	yes	gggaggugggugggggccu	gccccucccauaccua	gccccucccauaccuagugggaggugggugggggccu
chr9	140172186	140172253	6,8	57 +/- 7%	18	17	0	1	no	cggccgcucccuggggaccca	ggcccaggaggccccu	cggccgcucccuggggacccacaggugucgcugccccuccaggccgaagcuggcccaggaggccccu
chr14	106052329	106052378	6,8	74 +/- 6%	10	7	0	3	yes	caggccagugagacagggcc	cccucugcaugccucuggcu	caggccagugagacagggccugcccagggcccucugcaugccucuggcu
chr14	106172560	106172609	6,8	74 +/- 6%	10	7	0	3	yes	caggccagugagacagggcc	cccucugcaugccucuggcu	caggccagugagacagggccugcccagggcccucugcaugccucuggcu
chr1	28969555	28969605	6,8	37 +/- 23%	18	17	0	1	no	accggcugcugagacgaa	ccggaaguagucggggag	ccggaaguagucggggagggccgcgcagucggaccggcugcugagacgaa
chrX	148713146	148713206	6,7	63 +/- 12%	17	16	0	1	no	uucggcuccggggaggca	gcccccgggaguccgcg	gcccccgggaguccgcgcugcgggcucgggcgcgggcugguguucggcuccggggaggca
chr16	85045033	85045077	6,7	19 +/- 22%	17	16	0	1	no	gcgggacccaggggaccaagc	gcagccuccuuccgaccug	gcagccuccuuccgaccugcccggcgggacccaggggaccaagc
chr5	139018608	139018658	6,7	26 +/- 22%	10	8	0	2	yes	gaugggggcggaacagccucccuga	cacggaggcugggcug	gaugggggcggaacagccucccugacagauggacccacggaggcugggcug
chr12	121442296	121442362	6,7	37 +/- 23%	10	7	0	3	yes	gacagcgagaggcagcucugg	caggguacccucucguugca	gacagcgagaggcagcucuggggcuguguccugacuccagcuaacccaggguacccucucguugca
chr20	44054155	44054215	6,7	74 +/- 6%	18	14	0	4	no	ccgcucuuccccugaccccagg	augggggugagaugggggaggagcagc	augggggugagaugggggaggagcagccaguccugucucaccgcucuuccccugaccccagg
chr1	20047524	20047565	6,7	58 +/- 28%	17	16	0	1	yes	aaccggagaagaaaac	uuccucugagguuggaga	uuccucugagguuggagagaauuccaaccggagaagaaaac
chr6	2892079	2892127	6,6	19 +/- 22%	17	15	0	2	no	ggugcugcugccugacgacggcgu	cgccaggaaggagcug	cgccaggaaggagcugagccugcuggugcugcugccugacgacggcgu
chr20	37078079	37078149	6,5	61 +/- 27%	10	7	0	3	yes	gcugaaagccguuucccgugu	ugcuggcgcuuucacacacu	gcugaaagccguuucccguguuuggaggguucacaccugucccuuucaaaugcuggcgcuuucacacacu
chr16	3598217	3598270	6,5	74 +/- 6%	10	7	0	3	yes	acaugugucugugucugcccagc	agggccacauagucacauaguca	agggccacauagucacauagucacauagucacaugugucugugucugcccagc
chr18	684890	684970	6,4	47 +/- 14%	10	7	0	3	yes	agaggaucacugagcucaggagu	agcaugggcaacagagug	agaggaucacugagcucaggaguuggaggcugcagugagcuacaauaauaccacugcacuccagcaugggcaacagagug
chr11	100841607	100841665	6,3	74 +/- 6%	8	6	0	2	yes	aggcugaauucccagugagu	uucgcugggaauucagccucugc	aggcugaauucccagugagugccuuuugaguugcauucgcugggaauucagccucugc
chrX	53228069	53228142	6,2	74 +/- 6%	9	7	0	2	yes	ugccugcccucuuccuccagg	cuggaggaaguggggcaagaag	cuggaggaaguggggcaagaaggaggauggagagcugggccagacaugcucuugccugcccucuuccuccagg
chr8	42995502	42995539	6,2	74 +/- 6%	16	10	0	6	no	gucaccgccgccugaguc	cgcgggaggcggaggcg	gucaccgccgccugagucaccgcgggaggcggaggcg
chr2	11484709	11484777	6,2	57 +/- 7%	16	15	0	1	no	gagcuggcugagggacugca	gcggcgcgcggccgga	gcggcgcgcggccggagacgggcggccgcgcgggagugugugcgccgggagcuggcugagggacugca
chr10	74057783	74057859	6,2	31 +/- 20%	16	8	0	8	no	aguugagggauuagcgggu	cgcgauccuacgggcgca	aguugagggauuagcgggugacgcaucucucccagucccucauggagacccgcgcgcccgcgauccuacgggcgca
chr15	41591434	41591509	6,1	42 +/- 21%	10	8	1	1	yes	aggcugaaaaggcuggga	cagccucccaaguagc	cagccucccaaguagcuaggauuacaggcgcgcaccaccacgcucagccugauuucuaggcugaaaaggcuggga
chr11	67806336	67806390	6	31 +/- 20%	17	16	0	1	no	gagguugagcccaggcug	ccugaggcgcuuaaucccg	gagguugagcccaggcuguggccuggcggacggguccugaggcgcuuaaucccg
chr11	72499105	72499153	6	47 +/- 14%	17	16	0	1	no	cucugggaggcuuugu	ugggccugcuuggggaucc	cucugggaggcuuuguggccugagugcugugggccugcuuggggaucc
chr2	46129808	46129856	5,8	18 +/- 19%	10	7	0	3	yes	ggucugggagcuuggcuagg	gaacccagcccccaga	ggucugggagcuuggcuaggaagacuucugaagaacccagcccccaga
chr14	23342456	23342521	5,7	68 +/- 21%	8	4	0	4	yes	uguccuaccuuccucucuccagc	ggaaggggaaggggugg	ggaaggggaaggggugggaugcaaaggggcugucuacuacccguguccuaccuuccucucuccagc
chr7	65509827	65509901	5,7	49 +/- 13%	18	11	0	7	no	gccgggcacgguggcuca	aucacgaggucaggag	gccgggcacgguggcucaugccuguaaucccaucacuuugggaggcugaggcagguggaucacgaggucaggag
chr10	120819525	120819584	5,6	40 +/- 21%	13	11	0	2	yes	ugcugccagucuccuucagaca	auugaaggcuaauuagcaguau	auugaaggcuaauuagcaguauaacuauaaauaguaaugcugccagucuccuucagaca
chr6	27114993	27115050	5,6	61 +/- 24%	15	11	0	4	no	gcggguuggagccggcg	gccgagugcaccgccugcu	gccgagugcaccgccugcuccgcaaggguaauuaugccgagcggguuggagccggcg
chr4	71379890	71379939	5,5	49 +/- 13%	17	16	0	1	no	uggcucacugcagccug	gguggagugcagugucgc	gguggagugcagugucgcuauccuggcucuccuggcucacugcagccug
chr1	45285133	45285222	5,5	70 +/- 6%	23	21	0	2	no	acuggcuucuauuguuuauuucu	aaguuugcgugacaccuugu	aaguuugcgugacaccuuguuuugagcuacauuuggggguuuauuggcuucugcagugugcaguaagacuggcuucuauuguuuauuucu
chr12	113729333	113729415	5,4	54 +/- 23%	7	4	0	3	yes	uggcugcuucccuuggucuccagg	cggggccauggagcagccugug	cggggccauggagcagccuguguagacggggaccugcccugcaugggcacccccucacuggcugcuucccuuggucuccagg
chr8	28924988	28925048	5,4	70 +/- 6%	9	8	0	1	yes	guucacauauaaagaagugacu	acagcuucucuaugugugggauucg	acagcuucucuaugugugggauucguguaaaugcgaagaguucacauauaaagaagugacu
chrY	9930517	9930587	5,4	30 +/- 34%	16	15	0	1	no	ggccugguuaguacuuggaug	cccugaacgcacccgc	cccugaacgcacccgcuugccucugaucuguugaagcuaagcagagucgggccugguuaguacuuggaug
chr18	77153815	77153881	5,3	70 +/- 6%	14	7	4	3	no	cccagagcgcggccagcu	gcggccaccgcgggccc	cccagagcgcggccagcucgcccggccucugcgccccggcccggccucugcggccaccgcgggccc
chr2	175464724	175464803	5,2	40 +/- 21%	7	5	0	2	yes	ugcgcauuagauucucagagga	ccuuaugagaaucuaaugccuga	ugcgcauuagauucucagaggagcaagaauccuaaagugaacugugcaugcgagcuccuuaugagaaucuaaugccuga
chr7	5567538	5567598	5,2	40 +/- 21%	7	6	0	1	yes	uccaggguccucacugccugu	ucggcugugggguccugugguguguu	ucggcugugggguccuguggugugugggggagcugucacauccaggguccucacugccugu
chr8	107710060	107710130	5,2	57 +/- 7%	16	14	0	2	no	gugccgaaaggaugugggcc	gcucaaggauggcacg	gugccgaaaggaugugggccggucccagccuggacgcgagaacuuccagaacuggcucaaggauggcacg
chr19	54466601	54466646	5,1	68 +/- 21%	15	5	0	10	no	gcgugaggccgccgggg	cgaggcgccgccgcccc	gcgugaggccgccgggguccuucuucuccgaggcgccgccgcccc
